# Comparison of methods for pre-processing, exosome isolation, and RNA extraction in unpasteurized bovine and human milk

**DOI:** 10.1371/journal.pone.0257633

**Published:** 2021-09-30

**Authors:** Sanoji Wijenayake, Shafinaz Eisha, Zoya Tawhidi, Michael A. Pitino, Michael A. Steele, Alison S. Fleming, Patrick O. McGowan

**Affiliations:** 1 Center for Environmental Epigenetics and Development, Department of Biological Sciences, University of Toronto Scarborough, Toronto, Ontario, Canada; 2 Department of Cell and Systems Biology, University of Toronto, Toronto, Ontario, Canada; 3 Department of Nutritional Sciences, University of Toronto, Toronto, Ontario, Canada; 4 Translational Medicine Program, The Hospital for Sick Children, Toronto, Ontario, Canada; 5 Department of Animal and Poultry Science, University of Guelph, Guelph, Ontario, Canada; 6 Department of Psychology, University of Toronto, Mississauga, Mississauga, Ontario, Canada; 7 Department of Psychology, University of Toronto, Toronto, Ontario, Canada; 8 Department of Physiology, University of Toronto, Toronto, Ontario, Canada; George Mason University, UNITED STATES

## Abstract

Milk is a highly complex, heterogeneous biological fluid that contains non-nutritive, bioactive extracellular vesicles called exosomes. Characterization of milk-derived exosomes (MDEs) is challenging due to the lack of standardized methods that are currently being used for milk pre-processing, storage, and exosome isolation. In this study, we tested: 1) three pre-processing methods to remove cream, fat, cellular debris, and casein proteins from bovine milk to determine whether pre-processing of whole milk prior to long-term storage improves MDE isolations, 2) the suitability of two standard exosome isolation methods for MDE fractionation, and 3) four extraction protocols for obtaining high quality RNA from bovine and human MDEs. MDEs were characterized via Transmission Electron Microscopy (TEM), Nanoparticle Tracking Analysis (NTA), and western immunoblotting for CD9, CD63, and Calnexin protein markers. We also present an optimized method of TEM sample preparation for MDEs. Our results indicate that: 1) Removal of cream and fat globules from unpasteurized bovine milk, prior to long-term storage, improves the MDE yield but not purity, 2) Differential ultracentrifugation (DUC) combined with serial filtration is better suited for bovine MDE isolation compared to ExoQuick (EQ) combined with serial filtration, however both methods were comparable for human milk, and 3) TRIzol LS is better suited for RNA extraction from bovine MDEs isolated by EQ and DUC methods. 4) TRIzol LS, TRIzol+RNA Clean and Concentrator, and TRIzol LS+RNA Clean and Concentrator methods can be used for RNA extractions from human MDEs isolated by EQ, yet the TRIzol LS method is better suited for human MDEs isolated by DUC. The QIAzol + miRNeasy Mini Kit produced the lowest RNA yield for bovine and human MDEs.

## Introduction

Maternal milk is the primary nutritional source of newborn mammals. Mammalian milk is a highly complex and heterogeneous biofluid that contains protein, lipids, carbohydrates, minerals, vitamins, active enzymes, hormones, immune factors, and microbiota [1–4]. Mammalian milk is also biologically customized to fit the physiological, neurodevelopmental, and immune requirements of offspring as they age [3–6]. Human colostrum is a rich source of immunological components including immunoglobulin A (IgA), lactoferrin, leukocytes, and human milk oligosaccharides (HMOs), and is a vital source of early-life immune programming. Transition and mature milk are mainly tailored to meet the nutrient and energy demands of the growing offspring [7, 8]. Recently, maternal milk was found to contain functional microRNAs (miRNAs) encapsulated within protective lipid droplets, referred to as milk-derived exosomes (MDEs) [9–17]. MDEs (a subtype of extracellular vesicles (EVs)) range from 30–150 nm in size and were identified in humans, cows, rodents, goats, pigs, and marsupials [16, 18, 19]. MDEs are exclusively secreted from mammary gland epithelial cells (MECs), can travel across offspring’s intestinal endothelium post-ingestion into circulation, and are taken up by surrounding tissues [13].

The resilience of MDEs and the MDE cargo, consisting of microRNAs (miRNAs), small peptides, and lipids, to low pH and gastrointestinal digestion highlights the therapeutic and bioengineering potential of MDEs in translational medicine [20–22]. For instance, a recent study by Hock et al., (2017) reported that rat MDEs promote intestinal epithelial cell viability (IEC), proliferation, and increase cell activity. The findings also suggested the use of MDEs as a preventative for the treatment of necrotizing enterocolitis, a lethal intestinal disease that affects premature infants [23]. These findings were further confirmed by subsequent studies that examined the effects of bovine MDE supplementation in NEC development and progression [24] as well as malnutrition-induced intestinal damage [25]. Upon intake into cells, MDEs may release their cargo and regulate cellular functions of the recipient cells [9–13, 15, 16, 19, 26, 27]. Specifically, there is evidence that milk miRNAs, enclosed within MDEs, can induce post-transcriptional regulation of target mRNA in recipient tissues [10, 17, 19, 26, 28] and cross biological barriers *in vitro* and *in vivo* [13, 29]. However, findings in this area have not been entirely consistent and comparable, in part due to the divergent methods used for MDE processing, isolation, storage, and quantification [30].

There is an urgent need to establish standardized and reproducible methods to isolate and quantify MDEs. In 2014, the International Society for Extracellular Vesicles (ISEV) published a Position Editorial detailing the minimal requirements and recommendations for the identification and characterization of extracellular vesicles and their proposed functions [31]. However, the use of non-standardized techniques to isolate exosomes from milk continues to be a significant issue, and this is exacerbated by the high intra- and interspecific variability that naturally exist across milk samples. Moreover, there is limited consensus on the shelf life and pre-processing requirements for raw milk used in EV research. In particular, some studies report that changes in temperature and long-term storage of milk do not affect milk composition, integrity, and the final yield of isolated MDEs, while alternate studies have found that the recovery of MDEs is largely influenced by sample collection, the method, and time of pre-processing [11, 32–34]. Consequently, here we tested three pre-processing techniques on unpasteurized, whole bovine colostrum to determine whether removing cream, fat globules, cellular debris, and/or casein proteins prior to ultracold storage is required to obtain high quality MDEs or can the processing be done post long- term ultracold storage.

The purity of the isolated exosomes can vary due to the presence of contaminating particles, other EVs, viscosity of the sample, the presence of milk proteins, and nucleic acids that are often precipitated alongside MDEs [35–37]. Density gradient ultracentrifugation (DG-UC), where exosomes are separated based on size, mass, and density in a sucrose or iodixanol gradient, is considered the gold standard for exosome isolations [38]. However, critical drawbacks of this technique include the requirement of large sample volumes (range of milliliters to liters) [39, 40], vehicle damage or exosome aggregation [39, 41, 42] (especially exosomes originating from highly viscous solutions such as milk), standardization issues [21, 43], and lipoprotein contamination, where high density lipids (HDLs) will sediment alongside MDEs due to similar densities [40, 41]. DG-UC can also be highly labor-intensive, can take more than 24 h to complete, and is low in throughput [44, 45]. Differential Ultracentrifugation (DUC) [11, 13, 46–48] and commercially-available polymer-based precipitation techniques (i.e. ExoQuick (EQ) reagent) [12] are two alternate exosome isolation methods that are not overly labor intensive and do not require large starting volumes [35–37, 43, 49]. Both techniques can result in a relatively high exosome yield [42] and can be used to separate different EV populations from milk [39, 41]. Nonetheless, DUC and EQ methods are somewhat undervalued and underused in lactation research, when compared to DG-UC, immunoaffinity capture, microfluidics, and size-exclusion chromatography techniques. However, each of these methods have inherent pros and cons associated with exosome yield, purity, and level of contamination with other EVs and/or protein aggregates [50]. Therefore, a single exosome isolation method should not be used universally on all biofluids, rather sample type, starting volume, viscosity of the biofluid, and other technical considerations must be critically evaluated.

DUC consists of sequential centrifugation with progressively stronger centrifugal force and time that pellets cells, debris, and different EV types, where 2000 x g centrifugation pellets large EVs, 10–20,000 x g pellets microvesicles, and ~100,000 x g pellets exosomes [39–41, 51]. DUC is highly effective for the isolation of exosomes with greater quality and purity. However, ultra-high speeds and continuous handling of the samples can result in MDE degradation and low recovery rates [52]. Moreover, similar to UC and DG-UC, DUC also requires specialized equipment in the form of refrigerated ultracentrifuges and specialized rotors that can reach 100–150,000 x g speeds [21, 43]. In comparison, EQ precipitation has a high recovery rate, is a faster method, does not require a large sample volume [12, 21, 35, 36, 43, 47, 49], requires a basic centrifuge, and can produce higher RNA and miRNA yield with greater purity than other techniques [53]. Moreover, chemical precipitation methods effectively minimizes the co-precipitation of macromolecular proteins that can be present in biological fluids [52]. However, one of the major disadvantages is that EQ precipitation may co-precipitate lipoproteins and other non-specific EVs that can interfere with downstream applications [21, 35, 36]. Thus, rigorous assessment of the enriched fractions is recommended by ISEV when this method is employed [52]. Moreover, further studies are necessary to confirm the suitability of DUC and EQ isolation techniques in mammalian milk research, especially taking into account milk-specific handling and storage requirements, user expertise, and the suitability of the methods for downstream applications, including RNA and protein analysis.

In this study, we tested two exosome isolation methods, EQ and DUC, to isolate MDEs from frozen, unpasteurized bovine and human milk. Transmission electron microscopy (TEM), Nanoparticle Tracking Analysis (NTA), and western immunoblot were used to characterize the yield and purity of the isolated MDEs. Moreover, given that EQ and DUC may not efficiently remove contaminates from the exosome samples, additional fractionation [54] and/or filtration techniques are often combined to surpass this complication in serum and plasma samples [55]. Thus, we combined serial filtration steps to EQ and DUC methods to enhance the purity of the isolated MDEs.

Since the identification of RNA in exosomes in 2007 [20], numerous extraction methods have been used for exosome RNA profiling, including real time quantitative PCR (RT-qPCR), microarrays, and RNA sequencing. However, enrichment and molecular profiling of MDEs remain technically challenging [47] due to the variability in RNA extraction protocols and commercially available RNA extraction kits that are often utilized in EV research [56–58]. Here, we tested four MDE-based RNA extraction protocols: three commercially available kits 1) QIAzol + miRNeasy Mini Kit (Q), 2) TRIzol + RNA Clean and Concentrator Kit (Tri+RCC), 3) TRIzol LS + RCC (TLS+RCC) and an inhouse phenol-based extraction method, 4) TRIzol LS (TLS) [59], to identify a reproducible and optimal RNA extraction method that can be used to isolate high quality RNA (≥17 nucleotides) from bovine and human MDEs.

Bovine and human milk samples were processed and analyzed separately because comparing MDE isolation techniques and RNA extraction protocols across different milk types of various species was beyond the scope of this study. The overall aim of the current study was to compare methods of milk pre-processing, two fundamental MDE isolation techniques, and four RNA extraction protocols that are commonly used in the EV field to identify the most robust and reproducible techniques that can be used to standardize MDE research. Our results may be useful for the selection of purification methods in future studies using human and/or bovine milk where sample volumes are limited, samples are subjected to extended storage times, and are frozen immediately upon collection (e.g., human donor milk banks).

## Materials and methods

### Bovine milk collection and processing

Unpasteurized bovine colostrum was obtained from Loa-De-Mede Holsteins Farm (Oshawa, Ontario, Canada) from 3 healthy Holstein cows within 1 day postpartum. 100 mL of bovine colostrum/cow was collected into sterile, DNA/RNase-free conical tubes via hand milking, stored at 4°C and transported to the University of Toronto, Scarborough for analysis within 24 h. The bovine colostrum was collected by the commercial dairy farm as part of routine operations and a portion was donated for the study. All freshly collected bovine samples were pooled to remove variability in milk composition across dairy cows but processed separately to ensure independent sample extractions.

**Fig 1:**
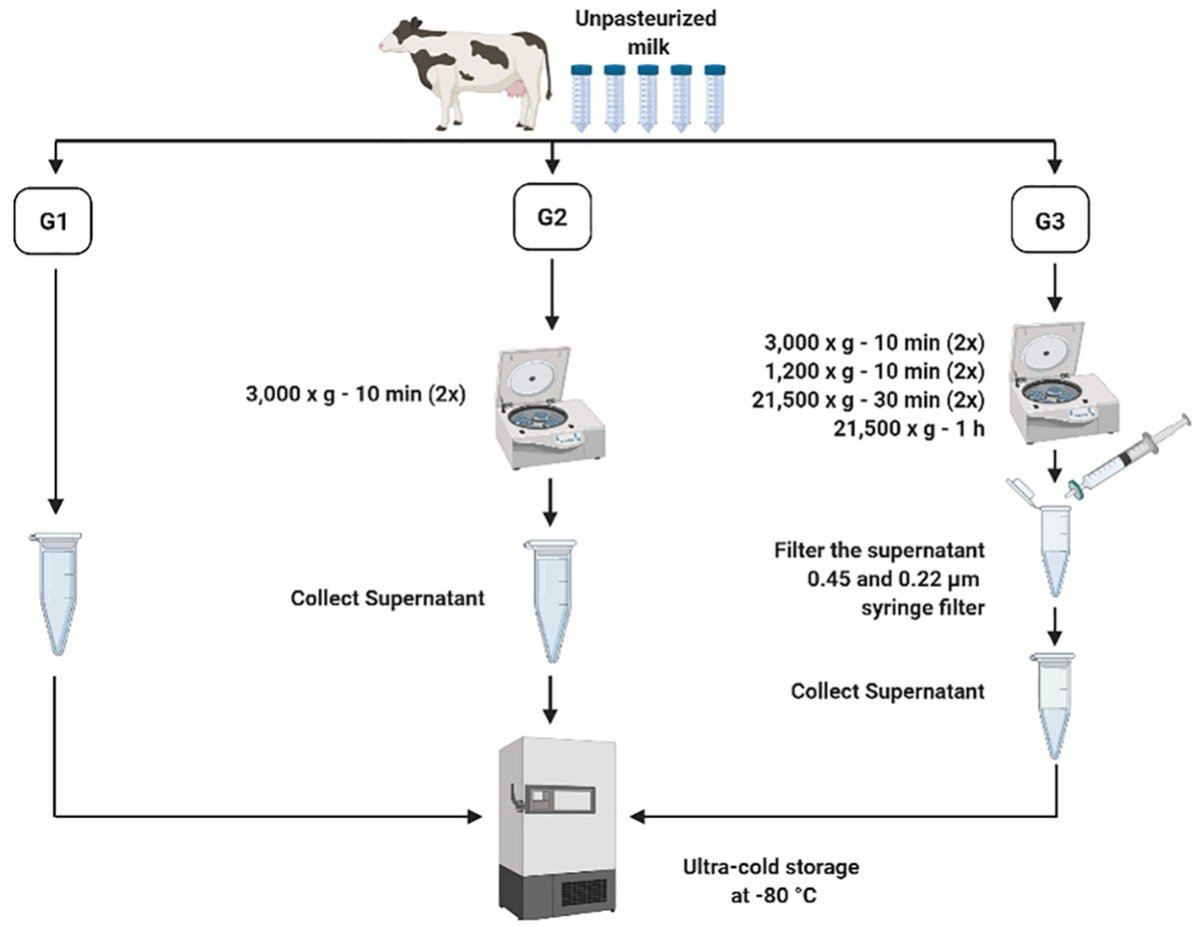
Pre-processing of bovine colostrum prior to long-term storage at -80°C. Group (G)1: whole milk frozen immediately upon collection and processed post-thaw. G2: Whole milk processed to remove fat globules and cream prior to ultracold storage. G3: Whole milk processed to remove fat globules, cream, milk cells, and casein proteins prior to ultracold storage.

Three pre-processing protocols were used to test whether removal of cream, fat globules, and casein proteins prior to long-term storage at -80°C may impact MDE isolation and characterization efficiency or post-freeze processing can be equally efficient at isolating MDEs with high quality and purity (n = 3 independent trials/group; Fig 1). Group (G) 1: unprocessed, whole milk stored immediately at -80°C upon arrival and processed to remove fat, cream, and casein proteins post long-term storage. G2: pre-processed milk without fat globules and cream, where bovine milk was centrifuged twice at 3,000 x g for 10 min at room temperature (RT) and the supernatant was collected and stored at -80°C. G3: isolated whey fraction without fat globules, cream, cellular debris, and casein proteins and stored at -80°C. G3 bovine milk samples were processed as per G2 procedure plus 2x centrifugations at 1,200 x g for 10 min at 4°C to remove residual fat globules and cellular debris. Subsequently, the defatted supernatants were centrifuged 2x at 21,500 x g for 30 min at 4°C followed by a subsequent centrifugation at 21,500 x g for 1 h to pellet casein proteins. The supernatants were filtered once through 0.45 μm (FroggaBio; SF0.45PES) and 0.22 μm (FroggaBio; SF0.22PES) PES syringe filters to remove residual cell debris. Isolated whey portion of bovine milk was stored at -80°C for later use. It should be noted that G1-G3 bovine milk samples were processed to remove lipids, cream, and casein proteins prior to MDE isolations. The difference across the three groups thus stems from the time at which the processing was conducted prior to long-term storage at -80°C.

### Human milk collection

Expressed human milk from 2 anonymous donors (500 mL/donor) were obtained from the Rogers Hixon Ontario Human Milk Bank (Toronto, Ontario, Canada). The unpasteurized samples of human milk used in this study contained a bacterial load >5 x 10^7^ colony forming units/L and were therefore not suitable for dispensing for neonatal consumption as per the policies of the milk bank. 500 mL of human milk/donor were collected into sterile collection bags, frozen at -20°C immediately upon collection, remained frozen during transport to the milk bank, and subsequently stored at -80°C till use. Next, the milk samples were thawed overnight at 4°C and were pooled but processed separately to ensure independent sample extractions. The samples were used in the analysis within 8 months of storage.

### Exosome isolation

**Fig 2:**
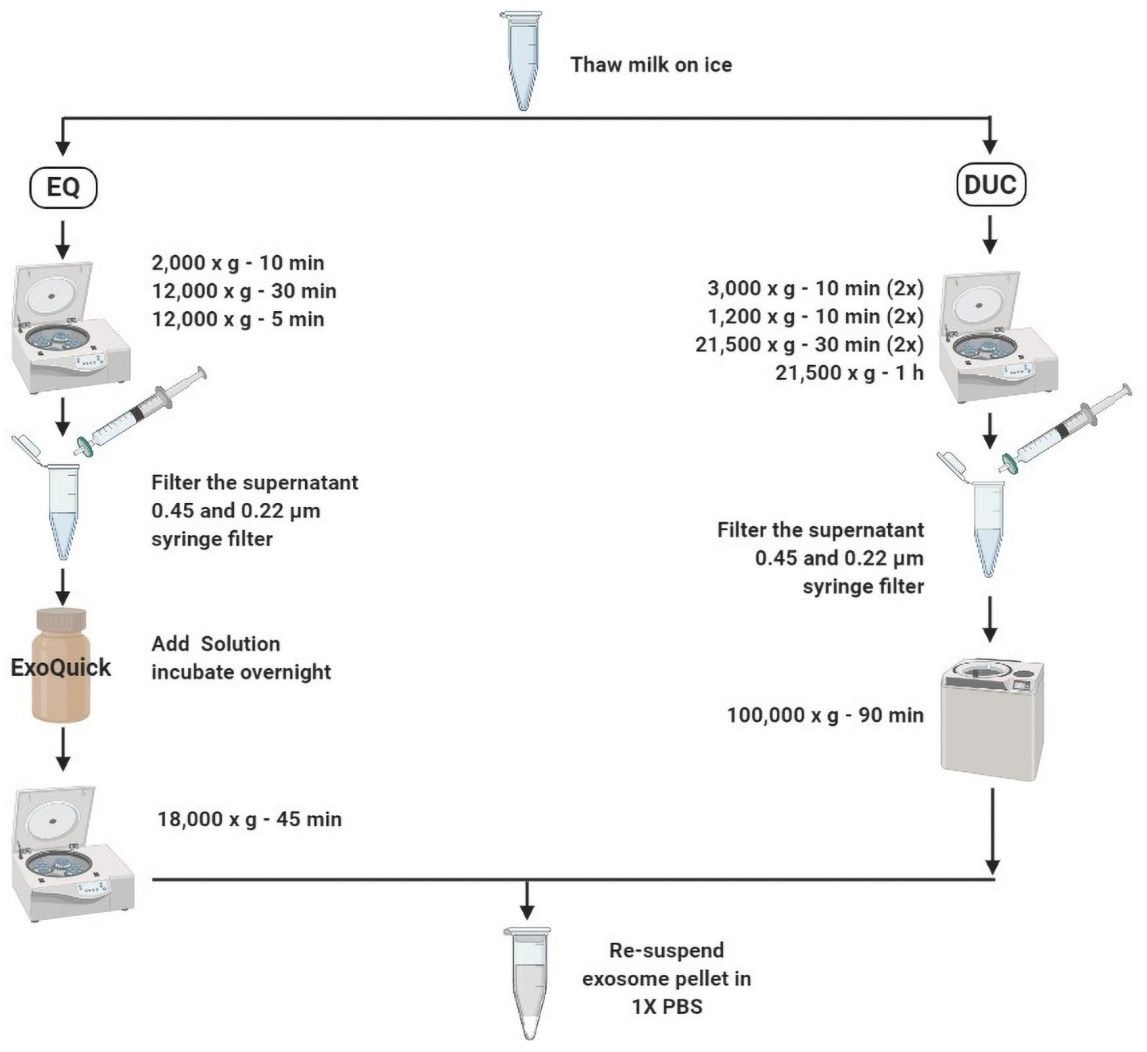
Bovine and human milk-derived exosome isolation via ExoQuick (EQ) precipitation and differential ultracentrifugation (DUC) methods.

Two isolation methods that are frequently used in exosome-isolation and characterization studies, including EQ precipitation and DUC, were compared to determine the most efficient method for isolating MDEs from the whey portion of unpasteurized bovine and human milk (n = 3 independent isolations/method) (Fig 2).

### Method 1: ExoQuick precipitation

The EQ precipitation method was used as previously described [12, 13, 49, 60, 61] to isolate MDEs from bovine G1-G3 and human milk using the EQ reagent (System Biosciences: EXOQ5A-1). Human and G1 bovine milk samples were thawed overnight at 4°C and centrifuged at 2,000 x g for 10 min at 4°C to remove upper cream layer. The supernatants were collected carefully and centrifuged again at 12,000 x g for 30 min at 4°C to remove fat cells and globules. Finally, the supernatants were further centrifuged at 12,000 x g for 5 min at 4°C to pellet cell debris. Supernatants were isolated and filtered once through 0.45 μm and 0.22 μm PES syringe filters to eliminate cellular debris. G2 bovine samples were thawed overnight at 4°C and filtered through 0.45 μm and 0.22 μm syringe filters to eliminate cellular debris. G3 bovine samples were thawed overnight at 4°C and were used directly in exosome precipitation. EQ reagent was added to all samples (1: 0.2, v/v), mixed by inversion, and incubated for 12 h at 4°C to enhance precipitation. Post incubation, all samples were centrifuged at 18,000 x g for 45 min at 4°C to pellet the exosomes. The pelleted exosomes were re-suspended in 200–400 μL of 1X-filtered PBS (determined based on the size of the pellet). The supernatants were used as the negative control for subsequent experiments.

### Method 2: Differential ultracentrifugation

DUC method was used as previously described [9, 10, 43, 60, 62, 63]. Human and G1 bovine milk samples were thawed overnight at 4°C and centrifuged twice at 3,000 x g for 10 min at RT to remove the upper cream layer. The supernatants were centrifuged twice at 1,200 x g for 10 min at 4°C, followed by two top-speed centrifugations at 21,500 x g for 30 min at 4°C and a final centrifugation at 21,500 x g for 1 h at 4°C to remove fat globules and casein proteins. The supernatants were filtered through 0.45 μm and 0.22 μm PES syringe filters to remove cell debris and residual fat cells. G2 bovine samples were thawed overnight at 4°C and centrifuged twice at 1,200 x g for 10 min at 4°C, followed by two top-speed, centrifugations at 21,500 x g for 30 min at 4°C and a final centrifugation at 21,500 x g for 1 h at 4°C to remove casein proteins. Similar to G1 bovine samples, the supernatants were filtered through 0.45 μm and 0.22 μm PES syringe filters to remove cell debris and residual fat cells. G3 bovine samples were thawed overnight at 4°C and directly used in the ultracentrifugation step. Finally, all whey fractions were centrifuged at 100,000 x g for 90 min using a SW55 Ti swing bucket ultracentrifuge at 4°C. The pellets were re-suspended in 200 μL of filtered 1X-filtered PBS. The supernatants were used as the negative control for all subsequent experiments.

### Exosome characterization

#### Transmission Electron Microscopy

The isolated MDEs were visualized by TEM with negative staining using an optimized sample preparation technique. Four-hundred mesh carbon-coated copper grids (Electron Microscopy Sciences; CF400-CU-50) were incubated for 5 min with 10 μL of isolated MDEs. Three consecutive wash steps with 20 μL of ddH_2_O (2 min each) were done to minimize crystallization and coagulation of milk residue. All copper grids were negatively stained with 10 μL of 2% uranyl acetate for 5 min at RT (S1 Fig). All excess reagents were removed with filter paper to ensure a 100 nm thickness and all grids were dried under an incandescent light for 10 min. The copper grids were observed and photographed using a Hitachi H-7500 transmission electron microscope with a Megaview III camera (Olympus). A magnification range of 80,000x – 120,000x were used for all samples. The level of magnification was determined based on the particle size, as MDEs can range from 30–150 nm in diameter within an isolate.

#### Nanoparticle tracking analysis

Particle size and concentration of isolated exosomes and negative controls were quantified using Nanoparticle Tracking Analysis (Malvern Instruments Ltd.; NanoSight NS300) as per manufacturer’s instructions at the Structural and Biophysical Core Facility, The Hospital for Sick Children (Toronto, Ontario, Canada). NTA uses the properties of Brownian motion and light scattering to measure particle size and concentration (particles/mL) of EVs. The software tracks individual particles frame by frame and calculates particle size based on Stokes-Einstein equation [64]. A 1:700 dilution factor for bovine samples and a 1:300 dilution factor for human samples were used for the analysis. Standard curves ranging from 1:100 to 1:700 (v/v in 1X-filtered PBS) were run per species to determine the optimal dilution range for the exosomes (60–100 particles/frame). An absolute control of 1X-filtered PBS was also assessed to test for the purity of the reconstitute media. Settings: detection threshold of 8, camera level of 15, 3 replicates of 30 s captures, and a blue laser 488 nm.

#### Western immunoblotting

Western immunoblotting was used to characterize the presence of exosome-specific, tetraspanin protein markers (CD9 and CD63) in the isolated MDE fractions. Exosome membranes are enriched in endosome-specific tetraspanins and, as such, CD9 (~28 kDa) and CD63 (~53 kDa) were used to test for the efficiency and purity of exosome isolations and the degree of cellular protein contamination. Calnexin (~68 kDa), an endoplasmic reticulum protein marker, was used as a negative cellular control to identify protein contamination resulting from non-exosomes and/or milk cells. Total soluble protein was isolated using phenol-based phase separation from 200 μL of isolated MDEs. Briefly, 100% ethanol was added (1:0.3, v/v) to the lower phenol phase incubated for 3 min at RT and centrifuged at 2,000 x g for 5 min at 4°C to pellet gDNA. Isopropanol (1:1.5, v/v) was added to the resulting supernatant and incubated for 10 min at RT. Subsequently, the samples were centrifuged at 12,000 x g for 10 min at 4°C to pellet the proteins. The pellets were washed with 0.3 M guanidine hydrochloride in 95% ethanol (1:2, v/v) and incubated for 20 min at RT. The washing step was repeated two more times. Finally, the pellets were washed with 2 mL of 100% ethanol and incubated for another 20 min at RT. All centrifugations were done at 7,500 x g for 5 min at 4°C. The pellets were air-dried for 10 min to remove residual ethanol and phenol contamination. The pellets were re-suspended in 200 μL of 1% SDS and incubated in a water bath at 50°C for 20 min. To enhance the solubility of proteins, the samples were incubated for 12 h at 4°C and centrifuged at 10,000 x g for 10 min at 4°C to remove insoluble material. Protein concentration was determined using a Pierce ^™^ Bicinchoninic Acid (BCA) Protein Microplate Assay (Thermo Scientific; 23225) and Bovine Serum Albumin (BSA) standards ranging from 2000 μg/mL to 0 μg/mL as per manufacturer’s instructions. Bovine G1-G3 MDE and human MDE samples were normalized to 4 μg/μL and mixed 1:1 (v/v) with 2X-SDS loading buffer (100 mM tris base, 4% (w/v) SDS, 20% (v/v) glycerol, 0.2% (w/v) bromophenol blue stain, 10% (v/v) 2-mercaptoethanol) to a final concentration of 2 μg/μL. Lastly, the samples were boiled for 10 min in a water bath, immediately cooled on ice, and stored at -20°C for later use.

8–10% SDS-polyacrylamide gels were used for CD9, CD63, and Calnexin protein quantification using a Mini-Protean III Electrophoresis apparatus (Biorad; 164–3301). 20 ug of bovine and human MDEs protein lysates were resolved/gel. 5μL of PiNK Plus Prestained Protein Ladder (Froggabio: PM005-0500, 10.5–175 kDa) was resolved/gel as a molecular weight reference and 20 μg of total soluble protein isolated from human microglia (ATCC: HMC3 cell line) was also resolved/gel as a cellular control. The gels were resolved for 45–75 min at 180 V in 1X tris-glycine running buffer (75.5 g of tris base, 460 g of glycine, 25 g of SDS, and ddH_2_O to add up to 2.5 L final volume) and electroblotted onto 0.45 μm polyvinylidene difluoride membrane (Millipore: IPVH00010) via a wet transfer system with 1X transfer buffer (60.6g tris base, 288 g glycine, 4 L methanol, 16 L of ddH_2_O) at 160 mA for 60–90 min. Membranes were blocked with 1–5% casein (v/v, 1X TBST) for 30–60 min, depending on the level of unspecific binding. After blocking, membranes were probed with primary antibody (1:1000, v/v, 1X TBST) at 4°C for 12 h for CD63 and Calnexin and 48 h for CD9. Finally, the membranes were incubated with goat HRP-conjugated anti-rabbit IgG secondary antibody (1:15,000, v/v, 1X TBST) for 45 min before visualization using enhanced chemiluminescence (H_2_O_2_ and luminol). Immunoblots were stained using Ponceau S Solution (Millipore-Sigma: P7170-1L) to correct for small discrepancies in protein loading. Antibodies used in this analysis include, CD9 (Systems Biosciences: EXOAB-CD9-1), CD63 (Systems Biosciences: EXOAB-CD63-1) and Calnexin (Genetex: GTX101676).

### RNA extraction from MDEs

**Fig 3:**
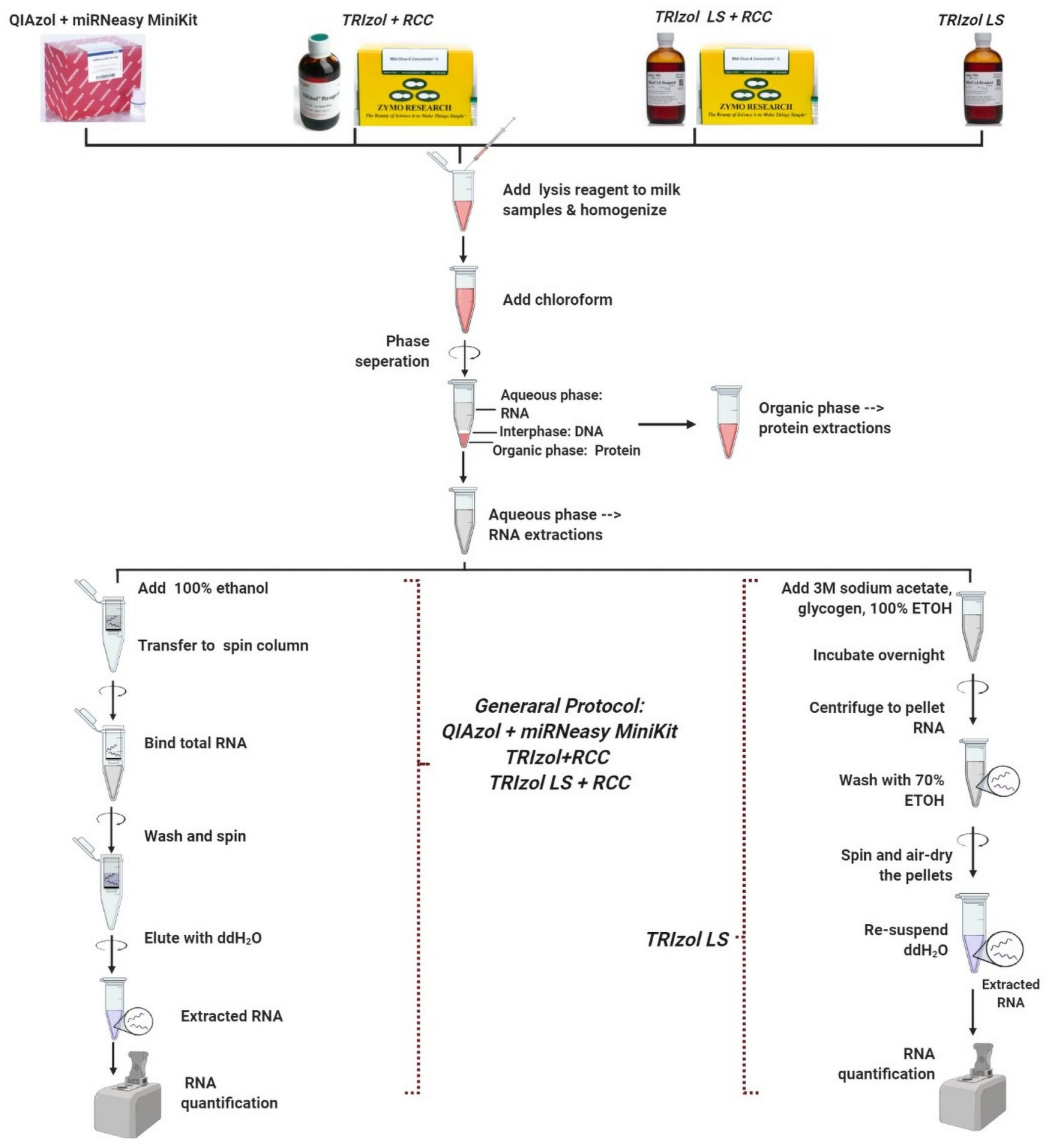
Four RNA extraction protocols. 1) QIAzol + miRNeasy MiniKit (Q), 2) TRIzol LS (TLS), 3) TRIzol + RNA Clean and Concentrator Kit (Tri+RCC), and 4) TRIzol LS + RNA Clean and Concentrator Kit (TLS+RCC) used for the isolation of total RNA from bovine and human milk exosomes.

Four RNA extraction methods that are commonly used in exosome studies (7–12,18,20,27,32,34,35,37,39,41,43–46) were compared to identify the most repeatable and suitable method to obtain high quality total RNA from MDEs isolated using EQ and DUC methods from unpasteurized bovine and human milk (n = 6 independent extractions/protocol; Fig 3).

#### Method 1: QIAzol + miRNeasy Mini Kit

QIAzol lysis reagent combined with miRNeasy Mini Kit (Qiagen; 217004) was used as per the manufacturer’s instructions with slight modifications. QIAzol reagent was added to 200 μL of isolated milk exosomes (5:1, v/v). All samples were homogenized by pipetting 20X followed by aspirating 20X with 18-gauge needles and incubated for 5 min at RT. Chloroform was added to each sample (1:1, v/v to the starting sample), then all samples were shaken vigorously for 15 s to mix and incubated for 3 min at RT. Phase separation was done by centrifuging at 12,000 x g for 15 min at 4°C. The upper aqueous phase was collected for RNA extraction and 100% ethanol (1.5:1, v/v) was added and pipetted to mix. The lower phenol layer was kept aside for protein extractions. All content, including any precipitate, was transferred to RNeasy mini columns, and centrifuged at 10,000 x g for 15 s at RT. Columns were washed according to the manufacturer’s instructions. Post washing, columns were centrifuged at the maximum speed for 5 min to remove ethanol contamination. RNA was eluted with 50 μL DNA/RNase free ddH_2_O. Columns were incubated for 10 min after adding DNA/RNase-free water and re-eluted to increase RNA yield. RNA concentration (ng/μL) and quality (A260/A280; A260/A230) were determined using a Nanodrop Spectrophotometer (Thermo Scientific; ND-2000C).

#### Method 2: TRIzol LS

TRIzol LS reagent (Thermo Scientific; 10296010) was used as described in [59] with minor modifications. Cold TRIzol LS reagent was added to 200 μL of isolated milk exosomes (3:1, v/v). All samples were homogenized by pipetting 20X followed by aspirating 20X with 18-gauge needles and incubated for 5 min at RT. 200 μL of chloroform was added to each sample. Samples were mixed by shaking for 30 s and incubated for 10 min at RT. Phase separation was done by centrifuging samples at 12,000 x g for 15 min at 4°C. The upper aqueous phase was collected for RNA extraction, while the lower phenol phase was kept aside for protein isolation. 10% sodium acetate (3M, pH 5.5), 4 μL of glycogen, and 100% ethanol (2.5:1, v/v) of the volume of aqueous phase were added per sample. Samples were mixed and incubated overnight at -80°C to facilitate RNA precipitation. Post incubation, samples were centrifuged at 16,000 x g for 30 min at 4°C to pellet the RNA. Subsequently, RNA pellets were washed with 500 μL of 70% ethanol and centrifuged at 16,000 x g for 5 min at 4°C. Ethanol was aspirated and pellet was centrifuged again at top speed for 1 min to remove any residual ethanol. Of note, this step was extremely important for the proper removal of ethanol contamination. The pellet was air-dried for 10 min and re-suspended in 32 μL of RNase-free ddH_2_O. RNA concentration (ng/μL) and quality (A260/A280; A260/A230) were determined using a Nanodrop Spectrophotometer (Thermo Scientific; ND-2000C).

#### Method 3: TRIzol + RNA Clean and Concentrator Kit

MDEs were lysed using TRIzol reagent (Thermo Scientific; 15596026) as per the manufacturer’s instructions with modifications. Cold TRIzol reagent was added to 200 μL of isolated MDEs (5:1, v/v). Samples were homogenized by pipetting 20X followed by aspirating 20X with 18-gauge needles and incubated for 5 min at RT. Subsequently, 200 μL of chloroform was added to the samples (0.2:1, v/v to TRIzol), vortexed for 30 s, and incubated for 3 min at RT. Samples were centrifuged at 12,000 x g for 15 min at 4°C to induce phase separation. The colorless, upper aqueous phase, containing total soluble RNA, was collected while the lower phenol phase was kept aside for protein isolation. Following TRIzol phase separation, 100% ethanol was added to the aqueous phase (1:1, v/v) and transferred to RNA Clean and Concentrator (RCC) ^™^ -5 kit (Zymo Research; R1013) and centrifuged at 16,000 x g for 30 s. RCC kit can be used to isolate ultra-pure, total RNA (≥17 ntd in length). Subsequently, the columns were washed once with RNA prep buffer and twice with RNA wash buffer (supplied with the kit) as per the manufacturer’s instructions. After the last wash, the columns were centrifuged on max speed for 5 min to remove residual ethanol. RNA was eluted with 40 μL of DNA/RNase-free water. Importantly, columns were incubated for 10 min after adding DNA/RNase-free water and re-eluted to increase RNA yield. RNA concentration (ng/μL) and quality (A260/A280; A260/A230) were determined using a Nanodrop Spectrophotometer (Thermo Scientific; ND-2000C).

#### Method 4: TRIzol LS + RNA Clean and Concentrator Kit

MDEs were lysed using TRIzol LS reagent (Thermo Scientific; 10296010) as per the manufacturer’s instructions with modifications. Cold TRIzol LS reagent was added to 200 μL of isolated milk exosomes (3:1, v/v). Samples were homogenized by pipetting 20X followed by aspirating 20X with 18-gauge needles and incubated for 5 min at RT. Subsequently, 200 μL of chloroform was added to the samples (0.3:1, v/v to TRIzol LS), vortexed for 30 s and incubated for 3 min at RT. Samples were centrifuged at 12,000 x g for 15 min at 4°C to induce phase separation. The colorless, upper aqueous phase, containing total soluble RNA was collected while the lower phenol phase was kept aside for protein isolation. All the steps involving the use of RCC kit are identical to that of TRIzol +RCC method. RNA concentration (ng/μL) and quality (A260/A280; A260/A230) were determined using a Nanodrop Spectrophotometer (Thermo Scientific; ND-2000C).

All RNA samples were separated via gel electrophoresis (1% TAE agarose, w/v) for 60 min at 300 mV to visualize RNA integrity and traces of cellular RNA contamination of the isolated exosome fractions. 100 bp DNA ladder (100 bp– 1,000 bp) (Genedirex: DM001-R500) and 1 kB DNA ladder (250 bp– 4,000 bp) (Genedirex; DM101-R500) and a cellular RNA control were run alongside the samples. RNA integrity profiles were generated using the BioAnalyzer 2100 (Agilent Technologies) at the Princess Margaret Genomics Center (University Health Network, Toronto, ON, Canada).

### Statistical analysis

Statistical analysis was conducted using SPSS statistical software (IBM Corp.), and figures were created using GraphPad Prism Version 7 and BioRender.com. A Shapiro-Wilk test was used to assess normality, as the sample size is less than n = 30/comparison. The data were normally distributed (p>0.05) and parametric analyses were carried out. SPSS boxplot outlier function was used to identify outliers with an interquartile range of (IQR) >3. A Two-way analysis of variance (ANOVA) was used to test for the main effects of bovine milk pre-processing (G1-G3), efficiency of exosome fractionation (pellet and supernatant), and their interaction for EQ and DUC isolation methods separately. A Two-way ANOVA was used to test for the main effects of exosome isolation methods (EQ and DUC), exosome fractionation, and their interactions in both bovine and human milk samples. Finally, a Two-way ANOVA was used to test for main effects of exosome fractionation, RNA extraction methods, and their interactions in bovine and human MDE samples. Tukey post-hoc analyses were used to conduct all pairwise comparisons. Relationships were considered statistically significant at *p* ≤ 0.05.

## Results

### Pre-processing of bovine milk

**Fig 4:**
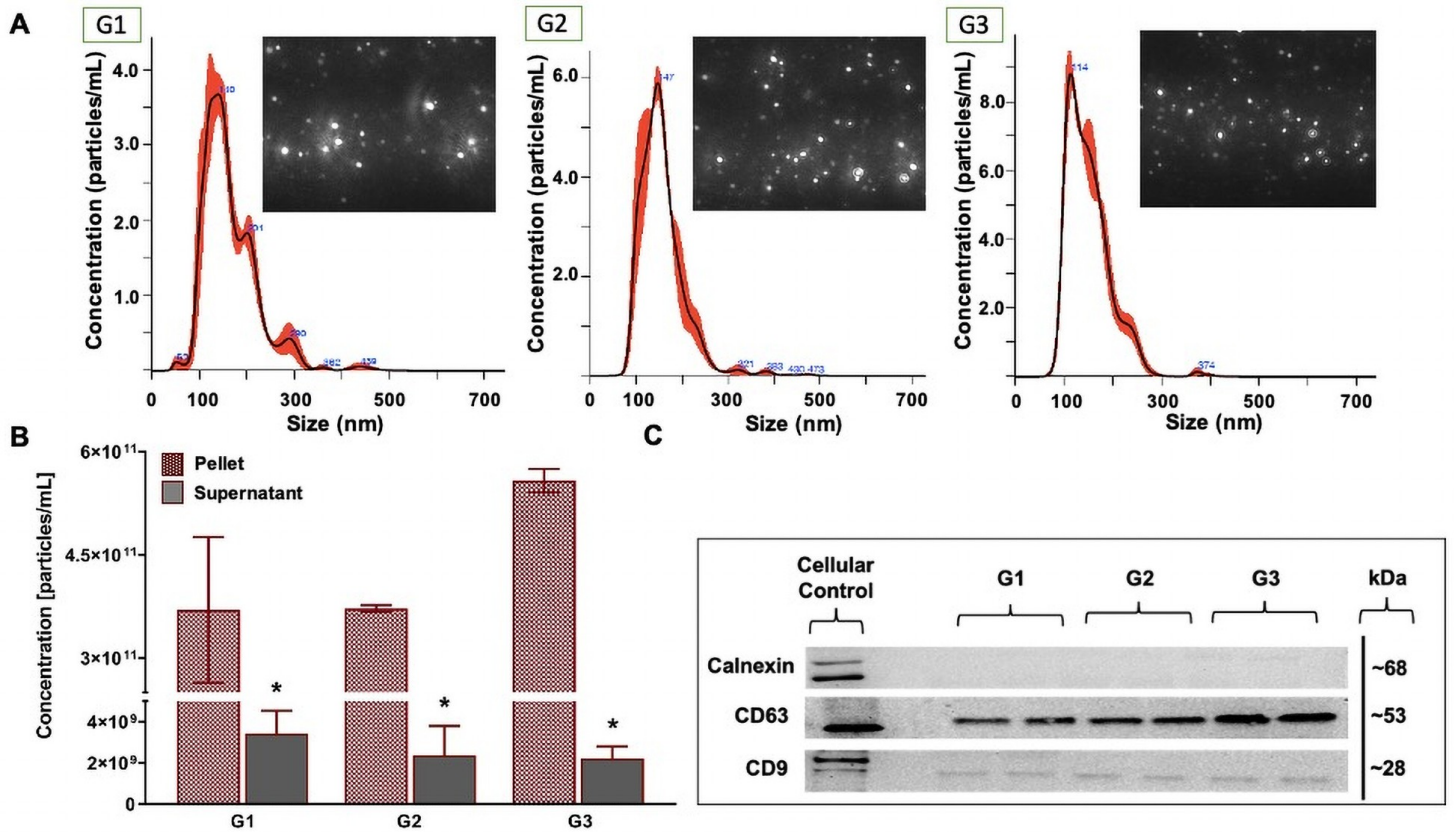
The effect of milk pre-processing prior to long-term storage for bovine milk-derived exosomes isolated via ExoQuick precipitation. Group (G)1: whole milk frozen immediately upon collection and processed post-thaw. G2: Whole milk processed to remove fat globules and cream prior to ultracold storage. G3: Whole milk processed to remove fat globules, cream, milk cells, and casein proteins prior to ultracold storage. Size and distribution profiles of bovine milk-derived exosomes as determined by Nanoparticle Tracking Analysis (NTA) (A). Concentration [particles/mL] of bovine milk-derived exosomes (B). Relative protein abundance of two exosome-specific markers (CD9 and CD63) and a cellular marker (Calnexin) as determined by western immunoblotting (C). Total soluble protein isolated from human microglia cell culture (HMC3) is used to represent cellular protein profiles. Data are mean ± SEM with n = 2 independent trials/group. * Significant difference in exosome concentration between the pellets and supernatants (p ≤ 0.05).

NTA results indicated no significant effect of pre-processing on bovine MDE concentration [particles/mL] when MDEs were isolated using the EQ method (main effect of pre-processing, (*F*_(2, 11)_ = 3.005, *p* = 0.125), Fig 4A and 4B) or particle size (F_(2,11)_ = 0.8778, *p* = 0.501, S2A Fig). The mean particle size of EQ-isolated bovine MDEs across the three groups was 132.67 ± 10.1. Protein abundance of exosome-specific markers, CD9 and CD63, generated single protein bands at 28 kDa and 53 kDa, respectively, across G1-G3 samples isolated with EQ method (Fig 4C). Calnexin, a mitochondrial protein marker, used in our study as a negative control to identify cellular contamination in the MDE isolations, failed to cross react at 68 kDa across G1-G3. Nevertheless, Calnexin strongly cross-reacted with the human microglia sample resolved alongside the isolated MDEs and generated a strong band at 68 kDa.

**Fig 5:**
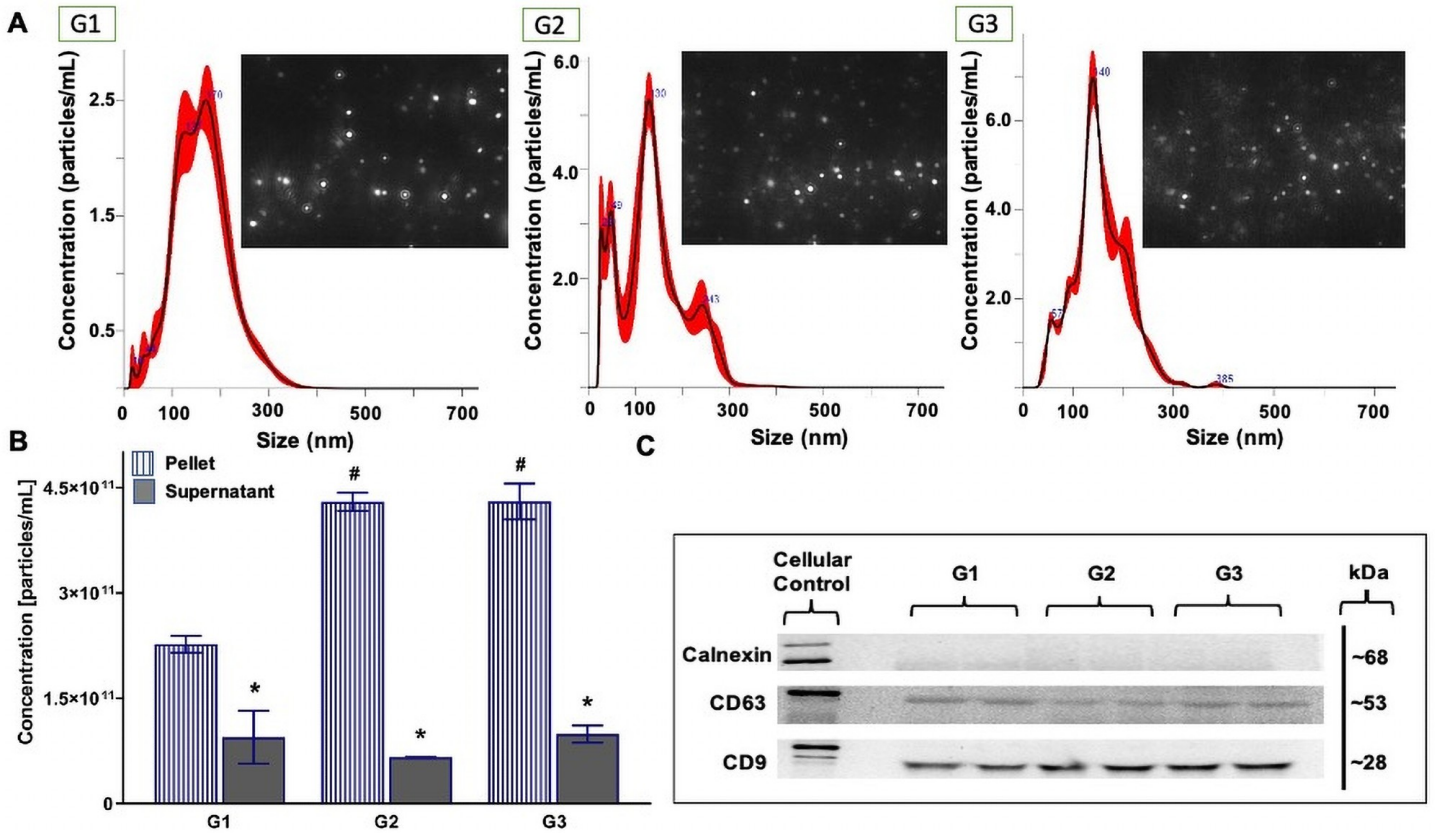
The effect of milk pre-processing prior to long-term storage for bovine milk-derived exosomes isolated via differential ultracentrifugation. Group (G)1: whole milk frozen immediately upon collection and processed post-thaw. G2: Whole milk processed to remove fat globules and cream prior to ultracold storage. G3: Whole milk processed to remove fat globules, cream, milk cells, and casein proteins prior to ultracold storage. Size and distribution profiles of bovine milk-derived exosomes as determined by Nanoparticle Tracking Analysis (NTA) (A). Concentration [particles/ml] of bovine milk-derived exosomes (pellet fraction) (B). Relative protein abundance of two exosome-specific markers (CD9 and CD63) and a cellular protein marker (Calnexin) as determined by western immunoblotting in the pellet fraction (C). Total soluble protein isolated from human microglia (HMC3) cells used to represent cellular protein profiles. Data are mean ± SEM with n = 2 independent trials/group. ^#^ Main effect of pre-processing (p ≤ 0.05). * Significant difference in exosome concentration between the pellets and supernatants (p ≤ 0.05).

There was a significant main effect of pre-processing when MDEs were isolated using DUC method (main effect of pre-processing, (*F*_(2, 11)_ = 14.816, *p* = 0.005), Fig 5A and 5B), where G1 isolations had significantly lower MDE yield [particles/mL], when compared to G2 (Tukey post hoc *p* = 0.003) and G3 (Tukey post hoc *p* = 0.003) isolations. Minimal differences in MDE yield were noted across G2 and G3 samples (Tukey post hoc *p* = 1.0). Minimal differences in particle size were recorded (F_(2,11)_ = 0.729, *p* = 0.552, S2B Fig) across the three groups. The mean particle size of DUC-isolated bovine MDEs across the three groups was 148.98 ± 8.55. In terms of the exosome purity across G1-G3 samples isolated via DUC, CD9 and CD63 exosome markers generated single protein bands at 28 kDa and 53 kDa, respectively, and Calnexin failed to cross react at 68 kDa (Fig 5C) in the MDE isolations yet generated a strong band in the human microglia sample. S3–S8 Figs show the complete immunoblot images of the protein targets.

**Fig 6:**
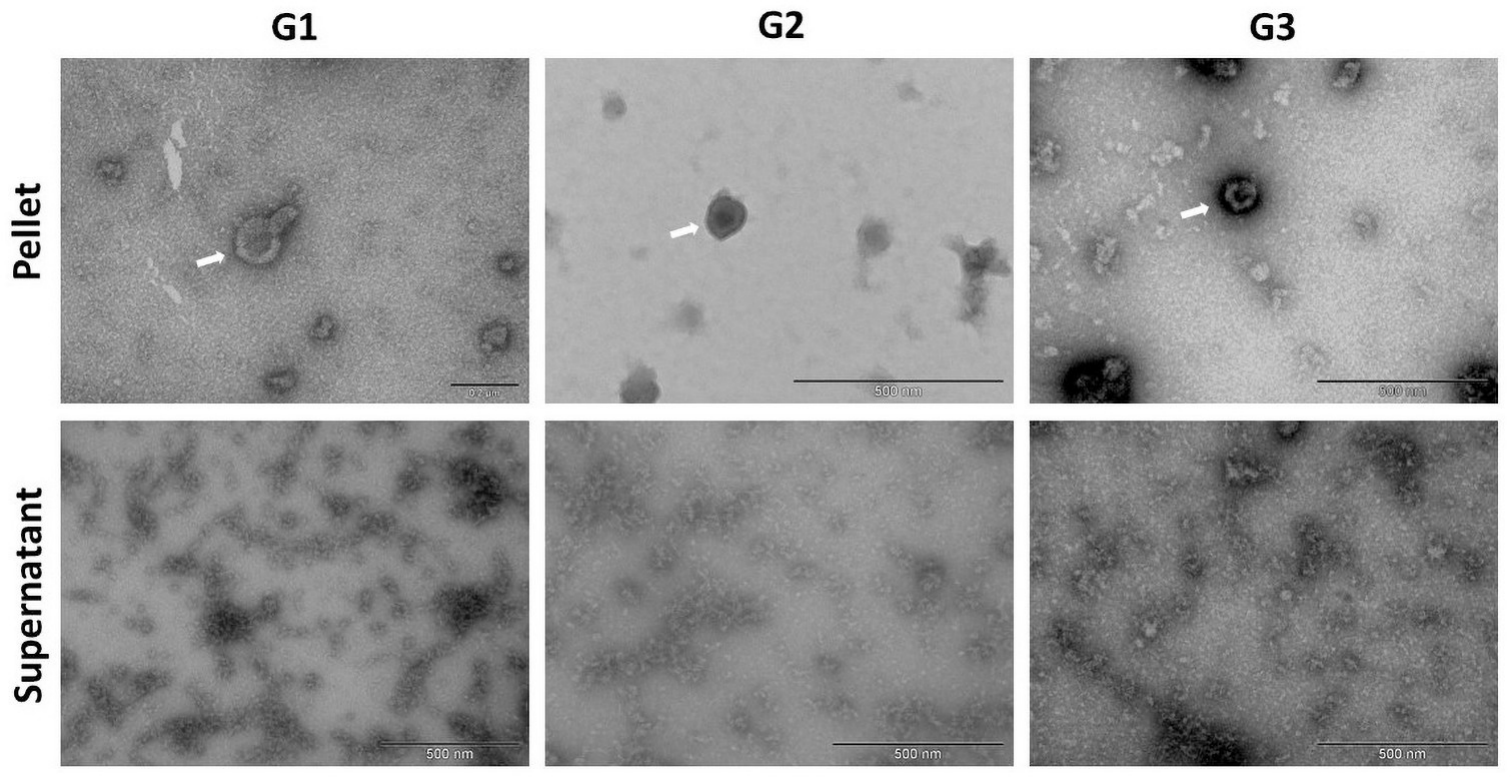
Morphology of bovine milk-derived exosomes isolated via the ExoQuick (EQ) method and visualized by Transmission Electron Microscopy (TEM). Group (G)1: whole milk frozen immediately upon collection and processed post-thaw. G2: Whole milk processed to remove fat globules and cream prior to ultracold storage. G3: Whole milk processed to remove fat globules, cream, milk cells, and casein proteins prior to ultracold storage. Scale bars: 200 nm—500 nm.

**Fig 7:**
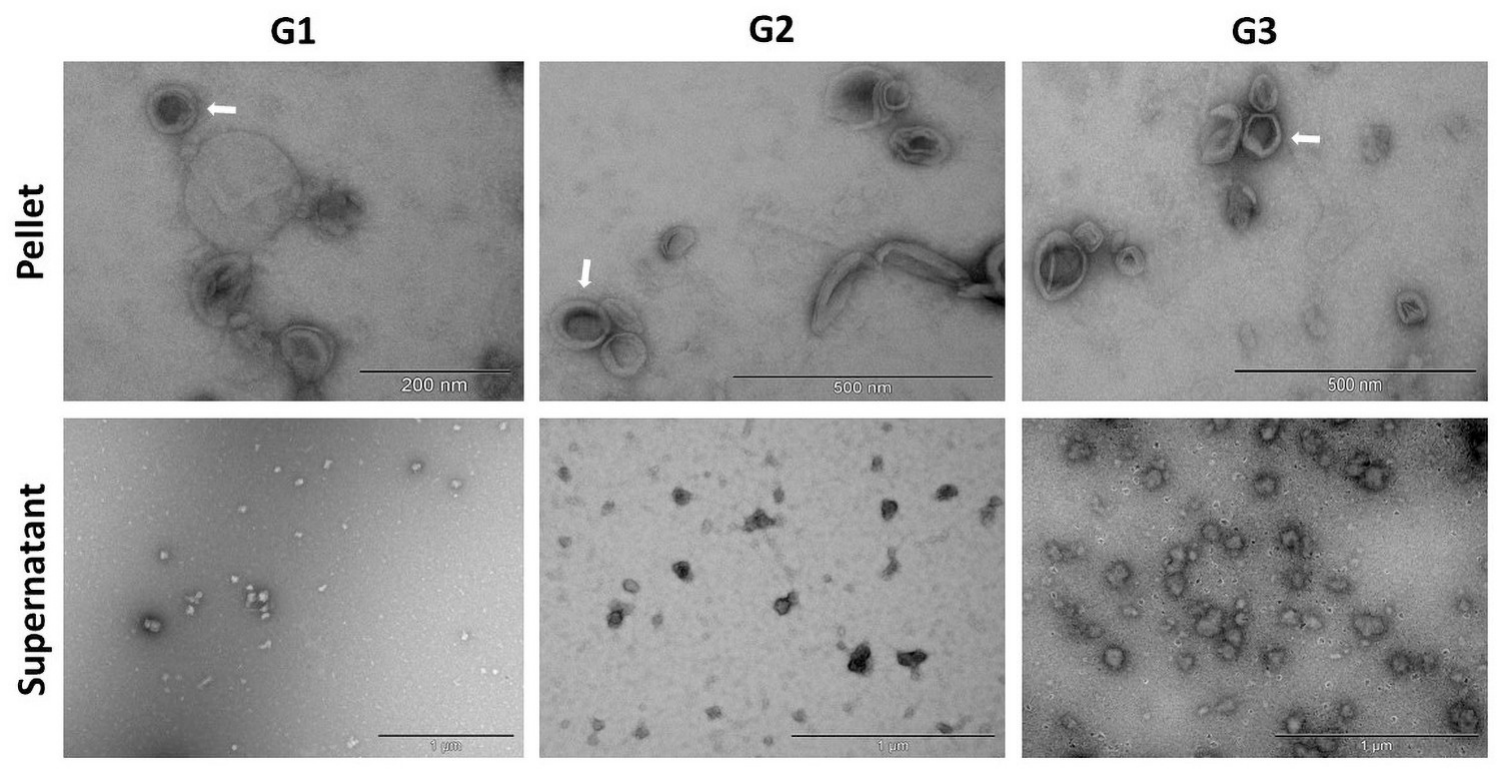
Morphology of bovine milk-derived exosomes isolated via differential ultracentrifugation (DUC) and visualized by Transmission Electron Microscopy (TEM). Group (G)1: whole milk frozen immediately upon collection and processed post-thaw. G2: Whole milk processed to remove fat globules and cream prior to ultracold storage. G3: Whole milk processed to remove fat globules, cream, milk cells, and casein proteins prior to ultracold storage. Scale bars: 200 nm—1000 nm.

Corresponding results were seen in TEM images of the EQ and DUC isolated MDEs, where G1-G3 EQ (Fig 6) and DUC (Fig 7) isolations illustrated minimal qualitative differences in morphology and purity of the MDEs. Specifically, similar levels of background cellular debris were noted across G1-G3 isolations, along with similar MDE morphology and distribution across pellet and supernatant.

### Bovine milk-derived exosome isolation: EQ versus DUC methods

Bovine MDE pellets were compared against their corresponding supernatants to test for efficiency of each fractionation, as supernatants should not contain appreciable amounts of MDEs. NTA results confirmed that G1-G3 exosome pellets contained significantly more MDEs [particles/mL], when compared to their respective supernatants for both EQ and DUC exosome isolation methods. EQ: (main effect of fractionation, (*F*_(1, 11)_ = 144.476, *p* < 0.001), Tukey post hoc G1: *p* = 0.008, G2: *p* = 0.008, G3: *p* = 0.001) and DUC: (main effect of fractionation, (*F*_(1, 11)_ = 271.474, *p* < 0.001), Tukey post hoc G1: *p* < 0.027, G2: *p* < 0.001, G3: *p* < 0.001). For DUC exosome isolation method, there was also a significant interaction between pre-processing group and fractionation (*F*_(2,11)_ = 18.633, *p* = 0.003). Similar results were seen in the TEM analysis, where EQ and DUC pellets contained more intact MDEs with the correct morphology, when compared to their respective supernatants. More cellular debris was present in the supernatant compared to the pellets.

EQ versus DUC isolation methods were compared to determine if one method is more suitable than the other for isolating high quality, intact MDEs from unpasteurized bovine colostrum. NTA results indicated minimal differences in MDE yield [particles/mL] across EQ and DUC methods (main effect of exosome isolation method, (*F*_(1, 23)_ = 0.038, *p* = 0.847)). Particle size (nm) also remained unchanged across EQ and DUC-isolated bovine MDEs (F(_1, 23_) = 3.688, *p* = 0.103). Correspondingly, CD9 and CD63 immunoblots illustrated uniform protein abundance in the EQ and DUC isolations, where strong protein bands were visible at 28 and 63 kDa, respectively, and calnexin failed to cross-react in the MDE samples, yet strongly cross-reacted in the cellular control. However, TEM illustrated qualitative differences in the morphology and the level of cellular debris between EQ and DUC isolations, where DUC pellets contained more intact, spherical exosomes compared to the EQ pellets. Some unidentified impurities and aggregates were observed in the EQ pellet fractionations.

### RNA extraction of bovine milk-derived exosomes

**Fig 8:**
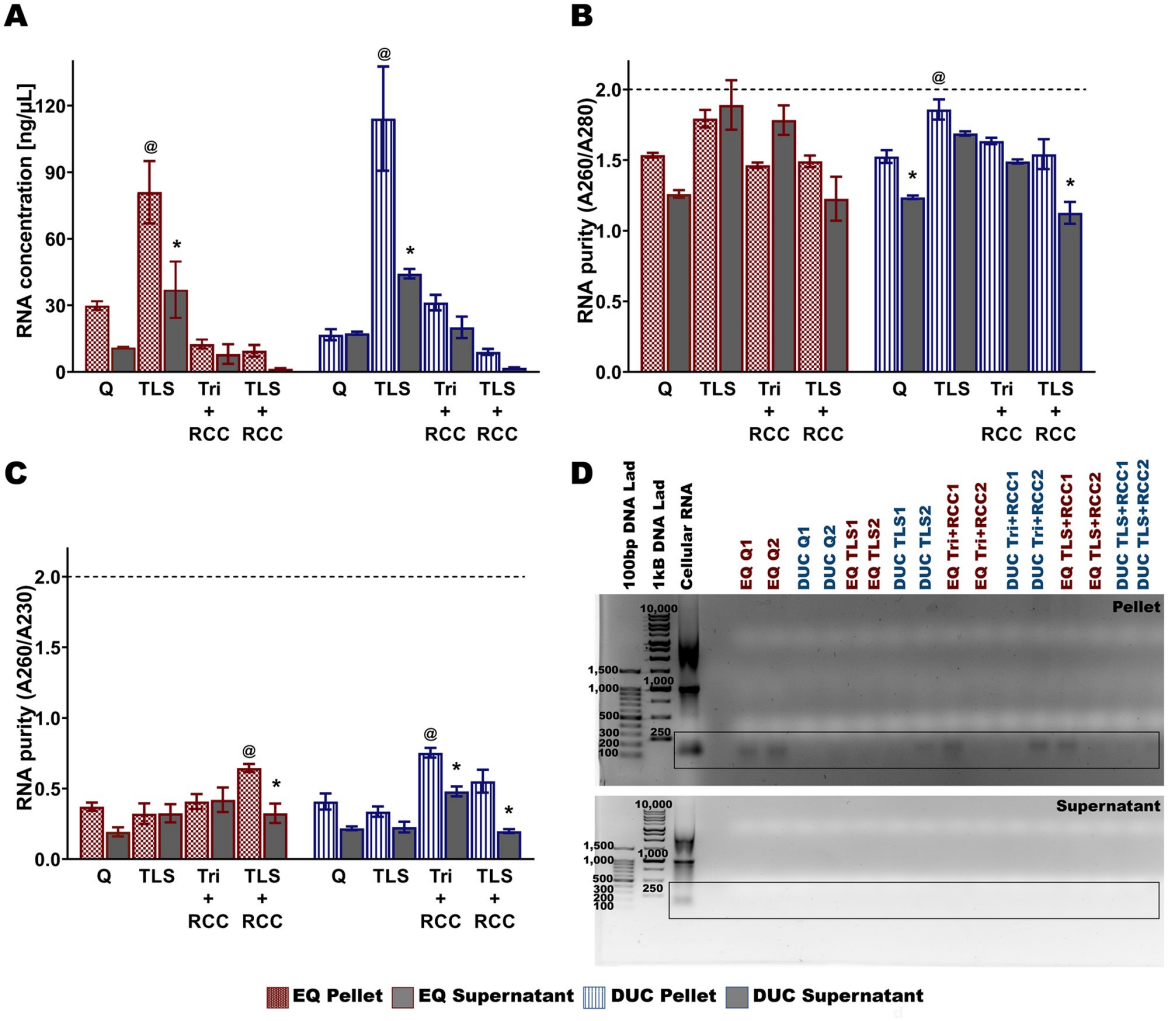
RNA yield [ng/μL], purity and quality of bovine milk-derived exosome pellets and supernatants isolated via ExoQuick (EQ) precipitation and differential ultracentrifugation methods (DUC). RNA was extracted using four protocols, 1) QIAzol + miRNeasy MiniKit (Q), 2) TRIzol LS (TLS), 3) TRIzol + RNA Clean and Concentrator Kit (Tri+RCC), and 4) TRIzol LS + RNA Clean and Concentrator Kit (TLS+RCC). RNA concentration [ng/μL] (A), RNA purity—absorbance at 260 nm/280 nm (B), and absorbance at 260 nm/230nm (C), and 1% TAE agarose gel electrophoresis (D) of the RNA samples. Data are mean ± SEM with n = 6 independent trials/group. ^@^ Main effect of RNA extraction protocol (p ≤ 0.05). * Significant difference in RNA concentration and purity between the pellets and supernatants (p ≤ 0.05).

The TLS protocol produced the highest RNA yield [ng/μL] for bovine MDEs isolated via EQ and DUC methods. EQ: TLS vs Q (main effect of RNA extraction protocol, (*F*_(3, 47)_ = 24.019, *p* < 0.001), Tukey post hoc *p* < 0.001, Fig 8A), TLS vs Tri+RCC (Tukey post hoc *p* < 0.001), and TLS vs TLS+RCC (Tukey post hoc *p* < 0.001). DUC: TLS vs Q (main effect of RNA extraction protocol, (*F*_(3, 47)_ = 28.408, *p* < 0.001), Tukey post hoc *p* < 0.001), TLS vs Tri+RCC (Tukey post hoc *p* < 0.001), and TLS vs TLS+RCC (Tukey post hoc *p* < 0.001). There was also a significant main effect of fractionation, (EQ: (*F*_(1, 47)_ = 14.488, *p* < 0.001, Tukey post hoc TLS: *p* = 0.002) and DUC: (*F*_(1, 47)_ = 12.772, *p* < 0.001, Tukey post hoc TLS: *p* < 0.001)) and significant fractionation/RNA extraction interaction, EQ: (*F*_(3, 47)_ = 3.227, *p* = 0.032) and DUC: (*F*_(3, 47)_ = 6.985, *p* = 0.001).

RNA purity (A260/A280) showed a significant main effect of RNA extraction protocol, (*F*_(3, 47)_ = 10.976, *p* < 0.001, Fig 8B) and fractionation/RNA extraction interaction, (*F*_(3, 47)_ = 4.643, *p* = 0.007) when MDEs were isolated using EQ method. For DUC MDE isolation method, TLS produced higher quality RNA (A260/A280) compared to Q (main effect of RNA extraction protocol, (*F*_(3, 47)_ = 25.003, *p* < 0.001), Tukey post hoc *p* = 0.004) and TLS+RCC (Tukey post hoc *p* = 0.007). There was also a significant main effect of fractionation (*F*_(1, 47)_ = 40.623, *p* < 0.001, Tukey post hoc Q: *p* = 0.017, TLS+RCC: *p* < 0.001) when MDEs were isolated via DUC method.

Moreover, TLS+RCC produced higher quality RNA (A260/A230) compared to Q (main effect of RNA extraction protocol, (*F*_(3, 47)_ = 4.892, *p* = 0.005), Tukey post hoc *p* = 0.037, Fig 8C) and TLS (Tukey post hoc *p* = 0.007) when MDEs were isolated using EQ method. There was also a significant main effect of fractionation (*F*_(1, 47)_ = 8.622, *p* = 0.005, Tukey post hoc TLS+RCC: *p* = 0.008) and significant fractionation/RNA extraction interaction (*F*_(3, 47)_ = 3.737, *p* = 0.019). For MDEs isolated using DUC method, Tri+RCC produced higher quality RNA compared to Q (main effect of RNA extraction protocol, (*F*_(3, 47)_ = 24.027, *p* < 0.001), Tukey post hoc *p* < 0.001), TLS (Tukey post hoc *p* < 0.001) and TLS+RCC (Tukey post hoc *p* = 0.043). There was also a significant main effect of fractionation (*F*_(1, 47)_ = 56.035, *p* < 0.001, Tukey post hoc Tri+RCC: *p* = 0.002 and TLS+RCC: *p* < 0.001) and significant fractionation/RNA extraction interaction (*F*_(3, 47)_ = 2.878, *p* = 0.048). Moreover, little to no ribosomal RNA contamination, in the form of 18S and 28S subunits, was present in the MDE samples. A single RNA in the range of 100–250 bp and RNA peaks in the range of 25 nt to <200 nt were visible in the 1% Agarose gel and BioAnalyzer profiles, respectively (Fig 8D, S13 Fig).

### Human milk-derived exosome isolation: EQ versus DUC methods

**Fig 9:**
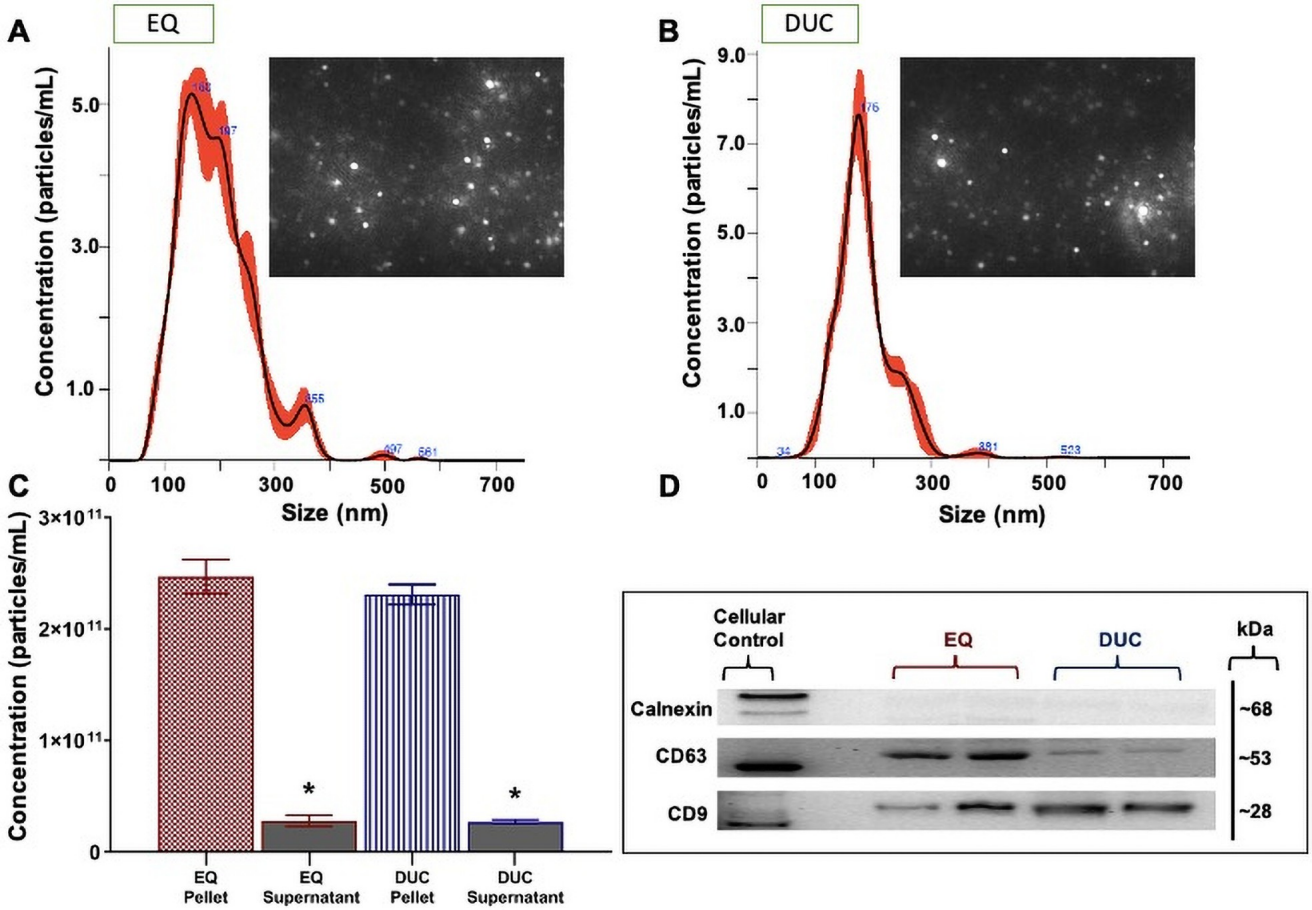
Human milk-derived exosomes isolated via ExoQuick (EQ) precipitation and differential ultracentrifugation (DUC) method. Size and distribution profiles of human milk-derived exosomes as determined by Nanoparticle Tracking Analysis (NTA) (A). Concentration [particles/mL] of human milk-derived exosomes (pellet fraction) (B). Relative protein abundance of two exosome-specific protein markers (CD9 and CD63) and a cellular protein marker (Calnexin) as determined by western immunoblotting in the pellet fraction (C). Total soluble protein isolated from human microglia cell culture (HMC3) is used to represent cellular total protein profiles. Data are mean ± SEM with n = 6 independent trials/group. * Significant difference in exosome concentration between the pellets and supernatants (p ≤ 0.05). S10–S12 Figs shows the complete immunoblot images of the protein targets.

Human MDEs isolated via EQ and DUC methods were compared against their corresponding supernatants to determine the efficiency of fractionation. NTA indicated that exosome pellets isolated via both EQ and DUC methods produced more MDEs [particles/mL], compared to their corresponding supernatants (main effect of fractionation, (*F*_(1, 7)_ = 534.670, *p* < 0.001), Tukey post hoc *p* < 0.001, Fig 9A and 9B). However, the NTA analysis comparing EQ and DUC indicated no differences in exosome yield [particles/mL] across the two isolation methods (main effect of exosome isolation method, (*F*_(1, 7)_ = 0.848, *p* = 0.409)). Particle size (nm) also remained unchanged across EQ and DUC (*t*_(1, 7)_ = 2.556, *p* = 0.237, S9 Fig), where the mean particle size of EQ-isolated human MDEs was 169.35 ± 0.07 and DUC-isolated human MDEs was 159 ± 5.79. Exosome-specific protein markers, CD9 and CD63, cross-reacted at 28 and 53 kDa, respectively, in EQ and DUC MDE fractions, and the cellular protein marker, Calnexin, failed to cross-react at 68 kDa in the MDE samples, yet strongly cross-reacted in the human microglia samples (Fig 9C).

**Fig 10:**
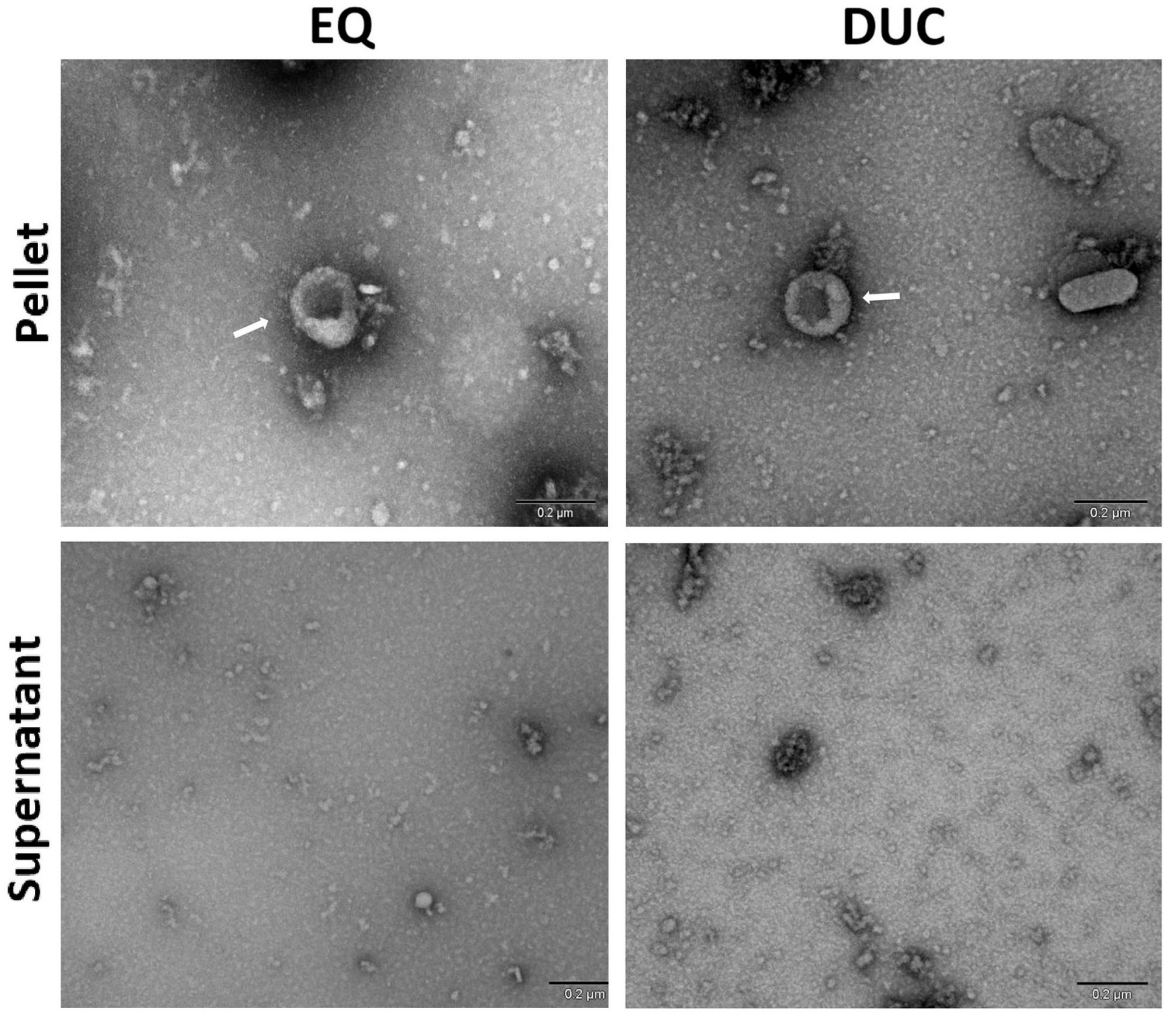
Morphology of human milk-derived exosomes visualized by Transmission Electron Microscopy (TEM) with negative staining (uranyl acetate). Human milk-derived exosomes were isolated via ExoQuick (EQ) precipitation and differential ultracentrifugation (DUC) methods. Scale bars: 200 nm.

TEM illustrated corresponding results, where EQ and DUC pellets contained more intact exosomes of the correct size compared to their respective supernatants, yet minimal differences in morphology and quality were observed in MDE pellets across EQ and DUC (Fig 10).

### RNA extraction of human milk-derived exosome

**Fig 11:**
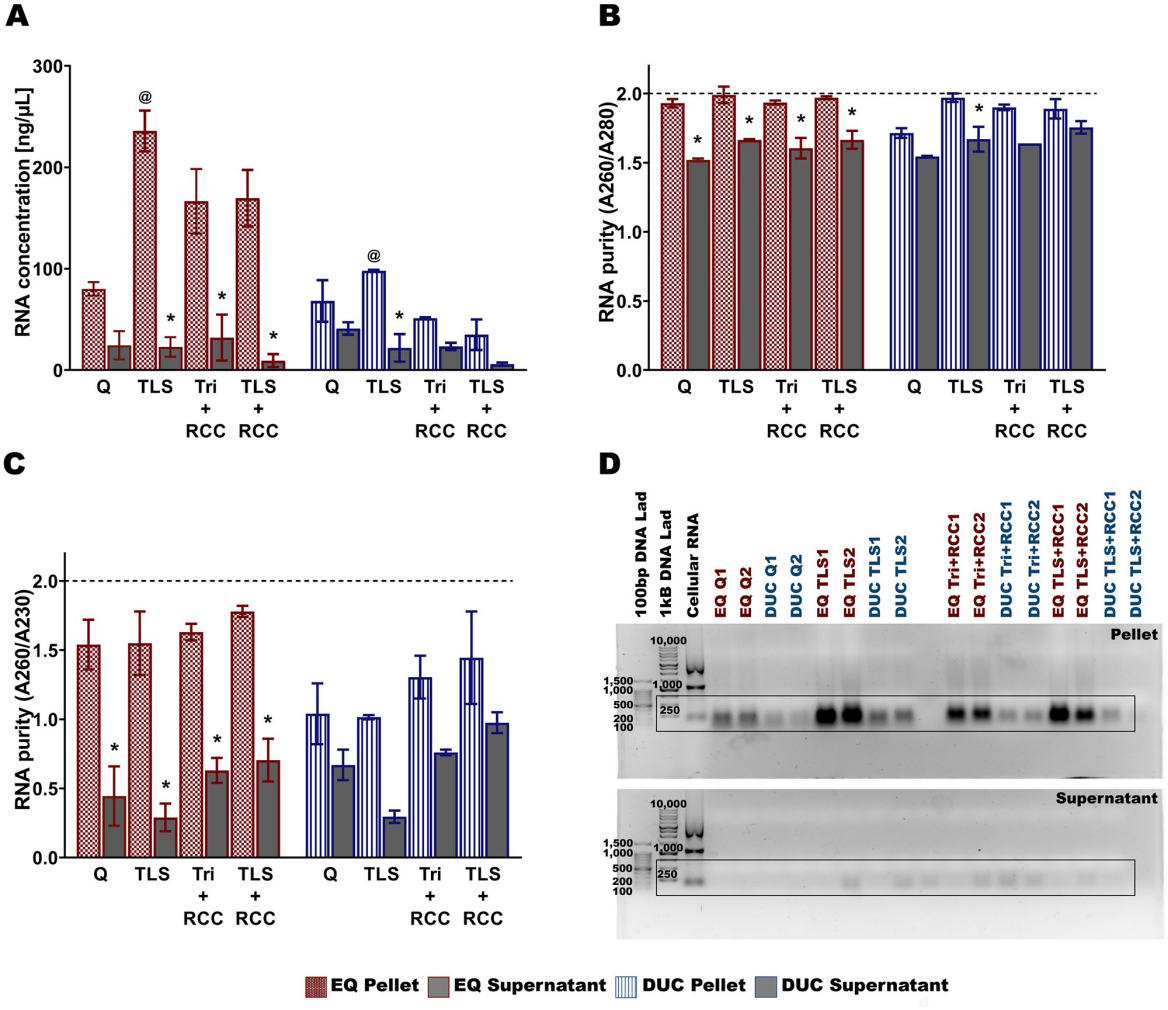
RNA yield [ng/μL], purity and quality of human milk-derived exosome pellets and supernatants isolated via ExoQuick (EQ) precipitation and differential ultracentrifugation methods (DUC). RNA was extracted using four protocols, 1) QIAzol + miRNeasy MiniKit (Q), 2) TRIzol LS (TLS), 3) TRIzol + RNA Clean and Concentrator Kit (Tri+RCC), and 4) TRIzol LS + RNA Clean and Concentrator Kit (TLS+RCC). RNA concentration [ng/μL] (A), RNA purity—absorbance at 260nm/280nm (B), and absorbance at 260nm/230nm (C), and 1% TAE agarose gel electrophoresis (D) of the RNA samples. Data are mean ± SEM with n = 3 independent trials/group. ^@^ Main effect of RNA extraction protocol (p ≤ 0.05). * Significant difference in RNA concentration and purity between the pellets and supernatants (p ≤ 0.05).

Minimal differences were seen across TLS, Tri+RCC, and TLS+ RCC extractions methods when MDEs were isolated using EQ method, however Q produced lower RNA yield [ng/μL] compared to TLS (main effect of RNA extraction protocol, (*F*_(3, 15)_ = 5.231, *p* = 0.027), Tukey post hoc *p* = 0.007, Fig 11A). There was a significant main effect of fractionation (*F*_(1, 15)_ = 5.231, *p* = 0.027, Tukey post hoc TLS: *p* = 0.001, Tri+RCC: *p* = 0.017 and TLS+RCC: *p* = 0.006) and significant fractionation/RNA extraction interaction (*F*_(3, 15)_ = 5.563, *p* = 0.023). For MDEs isolated via DUC method, TLS produced higher RNA concentration compared to TLS+RCC (main effect of RNA extraction protocol, (*F*_(3, 15)_ = 5.801, *p* = 0.021), Tukey post hoc *p* = 0.035). There was also a significant main effect of fractionation (*F*_(1, 15)_ = 28.800, *p* = 0.001, Tukey post hoc TLS: *p* = 0.012).

There was a significant main effect of RNA extraction protocol on RNA purity (A260/A280: (*F*_(3, 15)_ = 7.436, *p* = 0.011, Fig 11B); A260/A230: (*F*_(3, 15)_ = 4.419, *p* = 0.041, Fig 11C)) when MDEs were isolated using DUC method. There was also a significant main effect of fractionation (A260/A280: (*F*_(1, 15)_ = 42.573, *p* < 0.001, Tukey post hoc TLS: *p* = 0.025); A260/A230: (*F*_(1, 15)_ = 21.612, *p* = 0.002). For MDEs isolated via EQ method, there was only a significant main effect of fractionation (A260/A280: (*F*_(1, 15)_ = 126.818, *p* < 0.001, Tukey post hoc Q: *p* = 0.002, TLS: *p* = 0.009, Tri+RCC: *p* = 0.009 and TLS+RCC: *p* = 0.014); A260/A230: (*F*_(1, 15)_ = 109.728, *p* < 0.001, Tukey post hoc Q: *p* = 0.011, TLS: *p* = 0.005, Tri+RCC: *p* = 0.019 and TLS+RCC: *p* = 0.013). Similar to bovine MDE RNA profiles, human MDE RNA contained little to no ribosomal RNA contamination at 18 S and 28 S subunit range. A single RNA band and peaks in the range of 100–250 bp were visible in the 1% Agarose gel and BioAnalyzer profiles, respectively (Fig 11D, S14 Fig).

## Discussion

Recent studies have identified a number of novel non-nutritive bioactive components in maternal milk, including MDEs, milk miRNAs, and stem cells. MDEs and milk miRNAs are highly abundant across milk fractions from the milk fat globule membrane to whey fraction [16, 17, 26, 65], appear stable in the highly acidic and enzymatically rich environment of the gastrointestinal tract [10, 12, 66], and localize in peripheral organs [13], the blood [18], and the brain [29] of offspring upon consumption. These findings suggest a potential role for MDEs and milk miRNAs in postnatal development. As such, it is imperative that reproducible and standardized methods for milk collection, processing, storage, and MDE isolations are used across studies to ensure accurate characterization of MDEs and their biological function. Contradictory evidence exists on the necessity for unpasteurized milk pre-processing prior to ultracold storage, although in a majority of cases involving human donor milk and/or milk collected outside of clinical settings, it is not feasible to pre-process whole milk prior to cold storage. Moreover, DG-UC and size exclusion chromatography-based methods that are currently considered to be the gold standard for EV isolations have extensive limitations, as they require large sample volumes, can induce EV damage and/or aggregation and lipoprotein contamination, and can be highly labor and time-intensive. Therefore, alternate high throughput MDE isolation techniques that are less labor and time intensive, less damaging to the milk samples, and that can be scaled up or down to accommodate various volumes need to be evaluated for efficiency.

In this study, we tested three milk pre-processing methods, two standard MDE isolation techniques combined with serial filtration, and four RNA extraction protocols that can be used to obtain high quality exosome RNA from unpasteurized, bovine and human milk. Collectively, our results indicate that pre-processing of whole milk to remove milk fat globules and somatic milk cells affect the final MDE yield but not quality and purity when isolated with the DUC method. However, removal of casein protein and cellular debris from the milk fractions can be done post long-term ultracold storage without incurring significant loss in MDE yield. Moreover, we report that the EQ and DUC methods are useful for rapid isolation and increased MDE recovery from a small starting volume of bovine and human milk (1.5 mL). However, the quality and morphology of the MDEs isolated via the DUC method surpassed that of the EQ method for bovine colostrum. Of the four RNA extraction protocols tested on bovine MDEs isolated by EQ and DUC methods, the TLS protocol produced the highest RNA yield. TLS, Tri+RCC, and TLS+RCC methods were found to be efficient for the extraction of high-quality RNA from human MDEs isolated by the EQ method, yet TLS method was better suited for human MDEs isolated by DUC.

### Whole milk pre-processing

Some studies have reported that the pre-processing of whole, unpasteurized milk to remove fat, cream and unwanted protein and cellular debris prior to long-term storage affect the yield and the purity of isolated MDEs [32, 34], while other studies have reported minimal effects of milk pre-processing [11, 67, 68]. We found that ultracold freezing of unpasteurized bovine colostrum prior to the removal of milk fat globules, cream, and somatic milk cells, affect the final MDE yield, when MDEs were isolated via the DUC but not the EQ method (Figs 4 and 5). Indeed, cold storage of unprocessed breast milk at -80 °C has been shown to induce the death of somatic milk cells and lead to contamination of naturally present MDEs [32]. *In vitro* analysis has shown that disintegration of cell debris leads to the formation of membrane-enclosed vesicles that can interfere with EV pelleting [43]. As such, it is possible that increased apoptosis of somatic milk cells during 4 °C thawing may have interfered with the MDE pelleting via DUC, and a portion of MDEs may have been lost during the serial filtration and centrifugation steps. However, we note that all MDE isolates were enriched in CD9 and CD63 exosome-specific protein markers (Figs 4C and 5C), and the purity and morphology of MDEs did not vary across G1-G3 samples (Figs 6 and 7). In addition, there was no somatic milk cell contamination in the samples as evidenced by the lack of Calnexin protein abundance and ribosomal RNA contamination (18S and 28S subunits) in bovine MDE isolates (Fig 8D, S13 Fig). According to a study by Lasser et al (2011) that compared cellular RNA and exosome-specific RNA profiles across biofluids, exosome RNA has dissimilar BioAnalyzer profiles to that of cellular RNA. In particular, exosome RNA generally spans from >50 bp in length to approximately <300 bp, and does not contain peaks that correspond to 18S and 28S ribosomal subunits [46]. Therefore, our findings indicate that although ultracold storage of unpasteurized bovine colostrum without removing cream, fat globules, and somatic milk cells results in lower MDE yield, it does not greatly affect the purity or the morphology of the isolated MDEs. Interestingly, we also found that the removal of casein proteins and cellular debris via serial centrifugation and filtration prior to (G3) or post freezing (G2) generate similar MDE yields. Our findings are consistent with the findings of Howard et al., (2015) and Munagala et al., (2016), where pre-processing of whole milk prior to long-term storage did not enhance the integrity and/or biological activities of bioactive compounds [67, 68]. Extreme conditions such as hold pasteurization, involving 62.5 °C temperature treatment for 30 min, also does not appear to adversely affect the integrity and biological function of MDEs, and pasteurized MDEs are shown to be as functionally beneficial as raw milk MDEs [11]. Further, a majority of MDE research in human milk involves samples obtained from milk banks where it is frozen soon after expression without pre-processing. Thus, our findings demonstrating the ability to isolate high quality MDEs in appreciable quantities post ultracold storage is potentially of great value for research involving human milk MDEs. In addition, there is evidence that eliminating time sensitive pre-processing requirements soon after collection may increase the viability of other bioactive and macro/micronutrient components of milk, including hormones, vitamins, and growth factors, that are sensitive to repeated handling and temperature fluctuations [32, 34].

### Differential ultracentrifugation versus ExoQuick precipitation

UC-based isolations, EQ precipitation, size-based isolations, and immunity capture-based techniques are some of the most commonly used exosome isolation methods. Each technique utilizes a particular trait of EVs for the isolations, including density, shape, size, and/or surface receptors/membrane proteins. As such, there are unique advantages and disadvantages to each method [36]. DG-UC, where exosomes are separated based on size, mass, and density, is the gold standard for exosome isolations. However, DG-UC is not universally suitable for MDE isolations across milk fractions, types, and species due to differences in viscosity and composition of milk samples as well as practical and technical limitations surrounding large starting volumes, low throughput results, HDL contamination, and requirement of specialized equipment and prolonged processing times [40–42, 44, 51, 55]. As such, DUC and EQ precipitation coupled with serial filtration have become increasingly popular for rapid, large-scale bovine, human, and rodent MDE isolations in recent years [11, 12, 45, 47, 49, 61, 69].

We found that the EQ and DUC methods when combined with serial filtration and processing steps to remove fat, cream, and casein proteins, were equally efficient in isolating MDEs from 1.5 mL of unpasteurized bovine colostrum (Figs 4 and 5), where the pellets contained more intact MDEs compared to their respective supernatants. This was further corroborated by the TEM results, where a minimal number of intact bovine MDEs and higher portion of cellular debris were found in the supernatants (Figs 6 and 7), and the pellet and supernatant fractions did not contain somatic RNA (Fig 8D). Nevertheless, we did not find notable differences in using the EQ or DUC method to isolate bovine MDEs, as both isolation methods produced similar concentration of MDEs (NTA results) and the pellet fractions were enriched in CD9 and CD63, exosome positive protein markers, and failed to cross react with Calnexin, the cellular protein maker. However, we did observe differences in MDE morphology between the EQ and DUC methods, where DUC MDEs were more spherical and largely intact and the pellet fractions contained less cellular debris. Some unidentified structures were also observed in the EQ fractionations, suggesting that EQ pellet may contain protein impurities and/or other larger size EVs. A similar degree of impurity was also reported by Yamada et al., (2012) for bovine milk exosomes isolated with EQ reagent [49]. Moreover, Maburutse et al., (2017) reported that the use of EQ method for MDE precipitation from bovine colostrum lacks in specificity, where unwanted lipoproteins, protein: protein, and protein: RNA complexes are often co-precipitated [70]. The unspecific co-precipitation by EQ method has also been reported in other biological fluids, such as serum and plasma [42, 71], suggesting that co-precipitation of unwanted materials by commercial polymer-based precipitation solutions may not be unique to milk. However, the impurities and aggregates we noted are not cellular in origin as evident by the lack of Calnexin antibody cross reactivity and lack of ribosomal RNA profiles in EQ isolates.

Similar to that of bovine MDE isolations, the EQ and DUC methods resulted in the successful isolation of human MDEs of the correct particle size (30–150 nm), as evidenced by the higher concentration of exosomes in the pellet fractions compared to their respective supernatants (Fig 9A and 9B). We also observed minimal cellular protein (Fig 9C) and RNA contamination (Fig 11D), and enrichment of CD9 and CD63 exosome positive protein markers in the MDE pellets, confirming a high level of purity and exclusivity of the isolations. However, we did not observe differences in MDE yield, purity, and/or morphology across EQ and DUC methods for human milk. EQ has been shown to be a rapid and an acceptable method of MDE isolation by previous studies, as the polymer-based technology alters the solubility and dispersibility of the biological fluid and forces insoluble EVs out of the solution [35, 36, 53, 72]. Several maternal milk-based studies to date have used EQ method to successfully isolate MDEs in human and rodent milk [12, 45, 49, 69]. Nevertheless, it should be noted that EQ precipitation requires pre-clean up steps and may also co-precipitate non-exosome contaminants, including other unwanted EVs [36, 73]. As such, we added intermediary serial centrifugation and filtration steps (0.45 μm and 0.22 μm) to remove fat, cream, casein proteins, milk cells, cellular debris, and larger size EVs from the whey fractions prior to adding the EQ precipitation solution. Our findings indicate that these intermediary steps were important given that the human milk samples used in this study were obtained from a milk bank and were temporarily frozen upon collection at -20 °C and subsequently stored at -80 °C, which can interfere with polymer-based MDE isolations and ultracentrifugation-based methods [32].

Further, an interesting result to note is that the morphological differences in MDEs isolated via EQ and DUC methods were unique to bovine colostrum, whereas minimal differences were recorded in the TEM images of human MDEs across EQ and DUC. The differences in MDE morphology and the level of debris produced by EQ method may be attributed to the compositional and nutritional complexity of bovine colostrum compared to that of human donor milk. Although the exact lactation age of the human donor milk used in this study is unknown, it is not colostrum. Bovine colostrum is richer in lipids, peptides, non-protein nitrogen, vitamins, immune components, growth factors, hormones, and nucleotides, low in lactose [74], and enriched with MDEs [75], compared to transition and mature milk. As such, the biochemical and biophysical characteristics of bovine colostrum may have enhanced the existing disadvantages of EQ polymer-based reagent, and led to a greater co-precipitation of lipoproteins, other microvesicles, and contaminates. However, the compositional specificities of bovine colostrum that may have attributed to differences in TEM images remain to be investigated.

Based on our results, we conclude that a high MDE yield can be achieved with a small starting volume (1.5mL) of frozen bovine colostrum and human milk using both the EQ and DUC methods, whereas higher quality exosomes with intact morphological properties and less debris can be achieved by DUC exclusively for frozen bovine colostrum. Rapid and increased recovery methods are valuable for high throughput analysis of MDEs, especially if the milk samples are limited in quantity and there is limited access to specialized equipment and research expertise. This is a common reality in the developing world, as well as for lactation research conducted in field locations. Rapid isolation techniques may also enable more comprehensive analyses of RNA and protein kinetics and expression patterns to be characterized in MDEs [45, 49, 73] and preserve the functions and integrity of other non-nutritive bioactive compounds of milk [32, 34]. However, the use of DG-UC and size exclusion chromatography-based isolation techniques (SEC), and/or combination of DUC with SEC may be warranted for studies examining causal relationships between MDEs and physiological and/or disease states, clinical diagnostics, and for therapeutic interventions, due to the increased purity of MDEs that can be obtained with these techniques [76, 77]. Impurities and co-precipitation of unwanted aggregates and other microvesicles can produce confounding effects that interfere with downstream transcriptomics and proteomics analyses, *in vitro* and/or *in vivo* manipulation studies, functional assays, and impede biological interpretations related to milk composition and developmental impacts. Thus, researchers should choose the most suitable isolation method based on the nature of the biological fluid, sample volumes, downstream analyses/requirements, and the availability of resources and expertise.

### RNA extraction of bovine and human milk-derived exosomes

We found that the TLS protocol is best suited for extracting RNA from bovine MDEs isolated with EQ and DUC methods (Fig 8). This supports previous studies that have isolated high quality RNA suitable for high throughput sequencing from bovine MDEs using the Trizol LS protocol described herein [78, 79]. Moreover, minimal differences in RNA yield and/or purity across TLS, Tri + RCC, and TLS + RCC protocols were found for human MDEs isolated via EQ (Fig 11), suggesting that the three methods can be used to isolate good quality RNA from human EQ MDEs. Nevertheless, for human MDEs isolated via DUC method, TLS protocol was found to be most effective. The Q is the least preferable RNA extraction method, as this protocol produced the lowest RNA yield for bovine and human MDEs. For bovine and human MDEs, all four RNA extraction protocols yielded RNA ranging from 100–250 bp in size (1% Agarose gel). BioAnalyzer profiles of human and bovine MDE-RNA were also dissimilar to cellular RNA, where RNA peaks ranging from 25nt to <200nt were evident and peaks corresponding to 18s and 28S ribosomal RNA subunits were absent (S13 and S14 Figs). This indicates that the small non-coding RNA analyzed herein is of exosome-origin. Similar findings were reported in a previous study, where MDEs isolated from milk and other biological fluids contained dissimilar RNA profiles to that of cellular RNA, with little to no ribosomal RNA [46]. Nevertheless, our current data cannot differentiate MDE-RNA localized from within extracellular vesicles from non-vesicular RNA that may be associated with RNA Binding Proteins (RBPs) and/or lipoprotein contamination. RBPs are broadly defined as proteins that bind RNA through one or multiple globular RNA-binding domains (RBDs) and by doing so, determine the fate and function of the bound RNA [52]. Members of the RNA-induced silencing complex (RISC), specifically Argonaute-2 (Ago2), a key member of RISC, can be present in EV isolations and has garnered great interest due to conflicting reports associated with their cellular origins/localization [80]. For instance, several studies have presented compelling evidence for Ago2 and select RISC members to be included within EV subtypes, while others have indicated complete absence of Ago2-miRNA complexes in pure EV preparations [81–88]. Uncovering the location of Ago2-miRNA complexes in milk EVs has functional significance to understanding the mechanistic underpinnings of EV cargo-mediated transcriptional regulation of host cells and tissues, especially during early postnatal life in mammals. Although this is beyond the scope of our study, we encourage future works to utilize specialized methods, including immunoprecipitation using exosome-surface markers (CD9, CD63, CD81) and/or 2-dimentional gel electrophoresis combined with mass spectrometry and enzymatic (Proteinase K) and chemical digestion (detergents) methods to investigate the extracellular versus intracellular origins of Ago2 in MDE isolates.

## Conclusion

Overall, our findings illustrate that the removal of cream and fat globules prior to long-term storage is necessary to obtain a higher MDE yield, whereas the removal of casein protein and cellular debris can be conducted post-ultracold storage for unpasteurized bovine colostrum. Although outside the scope of the present investigation, further studies using additional bovine milk fractions, including milk fat globular membrane, as well as unpasteurized and unfrozen human milk is required to determine if these findings are transferable across milk types and species. Moreover, future work is necessary to determine if the decrease in MDE yield that we observed may be an indication of functional changes incurred by MDEs during the thawing process, where the lysing of milk cells and subsequent release of proteases and RNases may affect the integrity of MDEs and their cargo. We also found that the EQ and DUC methods are equally efficient for rapid isolation of MDEs from a small starting volume of bovine and human milk (1.5 mL), however the quality and morphology of the MDEs isolated via DUC surpassed that of the EQ method for bovine colostrum. These findings can be used to improve storage and rapid isolation methods for breast milk EVs. Due to the complexity and heterogeneity that naturally exists across milk types, subtypes, and fractions, researchers should choose an isolation method based on the nature of the biological fluid, starting volumes, downstream analyses/requirements, and the availability of resources and expertise.

## Supporting information

S1 Fig Optimized sample preparation protocol for Transmission Electron Microscopy (TEM) for the visualization of milk-derived exosomes. Copper grids were negatively stained with 2% uranyl acetate (UA).
(TIF)

S2 Fig Particle size distribution of bovine milk-derived exosomes isolated via ExoQuick precipitation. ExoQuick (A) and Differential Ultracentrifugation (B) as determined by Nanoparticle Tracking Analysis (NTA). Group (G)1: whole milk frozen immediately upon collection and processed post-thaw. G2: Whole milk processed to remove fat globules and cream prior to ultracold storage. **G3**: Whole milk processed to remove fat globules, cream, milk cells, and casein proteins prior to ultracold storage. Mean particle size is based on Stokes-Einstein equation with a 1:700 dilution in 1X-filtered PBS, 2 independent runs with 3 technical replicates of 30 s capture/run.
(TIF)

S3 Fig CD9 expression in bovine milk-derived exosomes isolated with ExoQuick protocol as determined by western immunoblotting. CD9 is presented as a positive, exosome marker and approximately 28 kDa in size. Lane 1: 1kB Pink Plus prestained protein ladder (range 10.5–175 kDa). Lane 2: Total soluble protein from human microglia (ATCC: HMC3 Cell line), used as the cellular control. Group 1: Frozen whole milk prior to processing. Group 2: Frozen milk without fat globules and cream. Group 3: Frozen whey fraction without fat globules, cream and casein proteins.
(PDF)

S4 Fig CD63 expression in bovine milk-derived exosomes isolated with ExoQuick protocol as determined by western immunoblotting. CD63 is presented as a positive, exosome marker and approximately 53 kDa in size. Lane 1: 1kB Pink Plus pre-stained protein ladder (range 10.5–175 kDa). Lane 2: Total soluble protein from human microglia (ATCC: HMC3 Cell line), used as the cellular control. Group 1: Frozen whole milk prior to processing. Group 2: Frozen milk without fat globules and cream. Group 3: Frozen whey fraction without fat globules, cream and casein proteins.
(PDF)

S5 Fig Calnexin expression in bovine milk-derived exosomes isolated with ExoQuick protocol as determined by western immunoblotting. Calnexin is presented as a negative, cellular marker. Lane 1: 1kB Pink Plus prestained protein ladder (range 10.5–175 kDa). Lane 2: Total soluble protein from human microglia (ATCC: HMC3 Cell line), used as the cellular control. Group 1: Frozen whole milk prior to processing. Group 2: Frozen milk without fat globules and cream. Group 3: Frozen whey fraction without fat globules, cream, and casein proteins.
(PDF)

S6 Fig CD9 expression in bovine milk-derived exosomes isolated with differential ultracentrifugation protocol as determined by western immunoblotting. CD9 is presented as a positive, exosome marker and is approximately 28 kDa in size. Lane 1: 1kB Pink Plus pre-stained protein ladder (range 10.5–175 kDa). Lane 2: Total soluble protein from human microglia (ATCC: HMC3 Cell line), used as the cellular control. Group 1: Frozen whole milk prior to processing. Group 2: Frozen milk without fat globules and cream. Group 3: Frozen whey fraction without fat globules, cream and casein proteins.
(PDF)

S7 Fig CD63 expression in bovine milk-derived exosomes isolated with differential ultracentrifugation protocol as determined by western immunoblotting. CD63 is presented as a positive, exosome marker and approximately 53 kDa in size. Lane 1: 1kB Pink Plus pre-stained protein ladder (range 10.5–175 kDa). Lane 2: Total soluble protein from human microglia (ATCC: HMC3 Cell line), used as the cellular control. Group 1: Frozen whole milk prior to processing. Group 2: Frozen milk without fat globules and cream. Group 3: Frozen whey fraction without fat globules, cream and casein proteins.
(PDF)

S8 Fig Calnexin expression in bovine milk-derived exosomes isolated with differential ultracentrifugation protocol as determined by western immunoblotting. Calnexin is presented as a negative, cellular control and is approximately 68 kDa in size. Lane 1: 1kB Pink Plus pre-stained protein ladder (range 10.5–175 kDa). Lane 2: Total soluble protein from human microglia (ATCC: HMC3 Cell line), used as the cellular control. Group 1: Frozen whole milk prior to processing. Group 2: Frozen milk without fat globules and cream. Group 3: Frozen whey fraction without fat globules, cream, and casein proteins.
(PDF)

S9 Fig Particle size distribution of human milk-derived exosomes isolated via ExoQuick precipitation and differential ultracentrifugation as determined by Nanoparticle Tracking Analysis (NTA). Mean particle size is based on Stokes-Einstein equation with a 1:500 dilution in 1X-filtered PBS, 2 independent runs with 3 technical replicates of 30 s capture/run.
(TIF)

S10 Fig CD9 expression in human milk-derived exosomes as determined by western immunoblotting. CD9 is presented as a positive, exosome marker and is approximately 28 kDa in size. Lane 1: 1kB Pink Plus pre-stained protein ladder (range 10.5–175 kDa). Lane 2: Total soluble protein from human microglia (ATCC: HMC3 Cell line), used as the cellular control. Lane 3–4: Milk exosomes isolated with ExoQuick protocol. Lane 5–6: milk exosomes isolated with the ultracentrifugation protocol.
(PDF)

S11 Fig CD63 expression in human milk-derived exosomes as determined by western immunoblotting. CD63 is presented as a positive, exosome marker and is approximately 53 kDa in size. Lane 1: 1kB Pink Plus pre-stained protein ladder (range 10.5–175 kDa). Lane 2: Total soluble protein from human microglia (ATCC: HMC3 Cell line), used as the cellular control. Lane 3–4: Milk exosomes isolated with ExoQuick protocol. Lane 5–6: milk exosomes isolated with the ultracentrifugation protocol.
(PDF)

S12 Fig Calnexin expression in human milk-derived exosomes as determined by western immunoblotting. Calnexin is presented as a negative, cellular control and is approximately 68 kDa in size. Lane 1: 1kB Pink Plus pre-stained protein ladder (range 10.5–175 kDa). Lane 2: Total soluble protein from human microglia (ATCC: HMC3 Cell line), used as the cellular control. Lane 3–4: Milk exosomes isolated with ExoQuick protocol. Lane 5–6: milk exosomes isolated with the ultracentrifugation protocol.
(PDF)

S13 Fig Bovine milk-derived exosome pellets isolated via ExoQuick (EQ) precipitation and differential ultracentrifugation methods (DUC) analyzed via BioAnalyzer 2100. RNA was extracted using four protocols, 1) QIAzol + miRNeasy MiniKit (Qiazol), 2) TRIzol LS (TLS), 3) TRIzol + RNA Clean and Concentrator Kit (Tri+RCC), and 4) TRIzol LS + RNA Clean and Concentrator Kit (TLS+RCC). All samples exhibit RNA profiles in the range of 25–200 bp, indicative of 5S and 5.8S subunits, tRNAs, and small RNAs and little to no 18S and 28S ribosomal RNA subunits.
(TIF)

S14 Fig Human milk-derived exosome pellets isolated via ExoQuick (EQ) precipitation and differential ultracentrifugation methods (DUC) analyzed via BioAnalyzer 2100. RNA was extracted using four protocols, 1) QIAzol + miRNeasy MiniKit (Qiazol), 2) TRIzol LS (TLS), 3) TRIzol + RNA Clean and Concentrator Kit (Tri+RCC), and 4) TRIzol LS + RNA Clean and Concentrator Kit (TLS+RCC). All samples exhibit RNA profiles in the range of 25–200 bp, indicative of 5S and 5.8S subunits, tRNAs, and small RNAs and little to no 18S and 28S ribosomal RNA subunits.
(TIF)
