# Supplementary figures and images for: Comparison of methods for pre-processing, exosome isolation, and RNA extraction in unpasteurized bovine and human milk
Source: PLoS One. 2021 Sep 30;16(9):e0257633. doi: 10.1371/journal.pone.0257633 (PMC8483318; doi:10.1371/journal.pone.0257633)

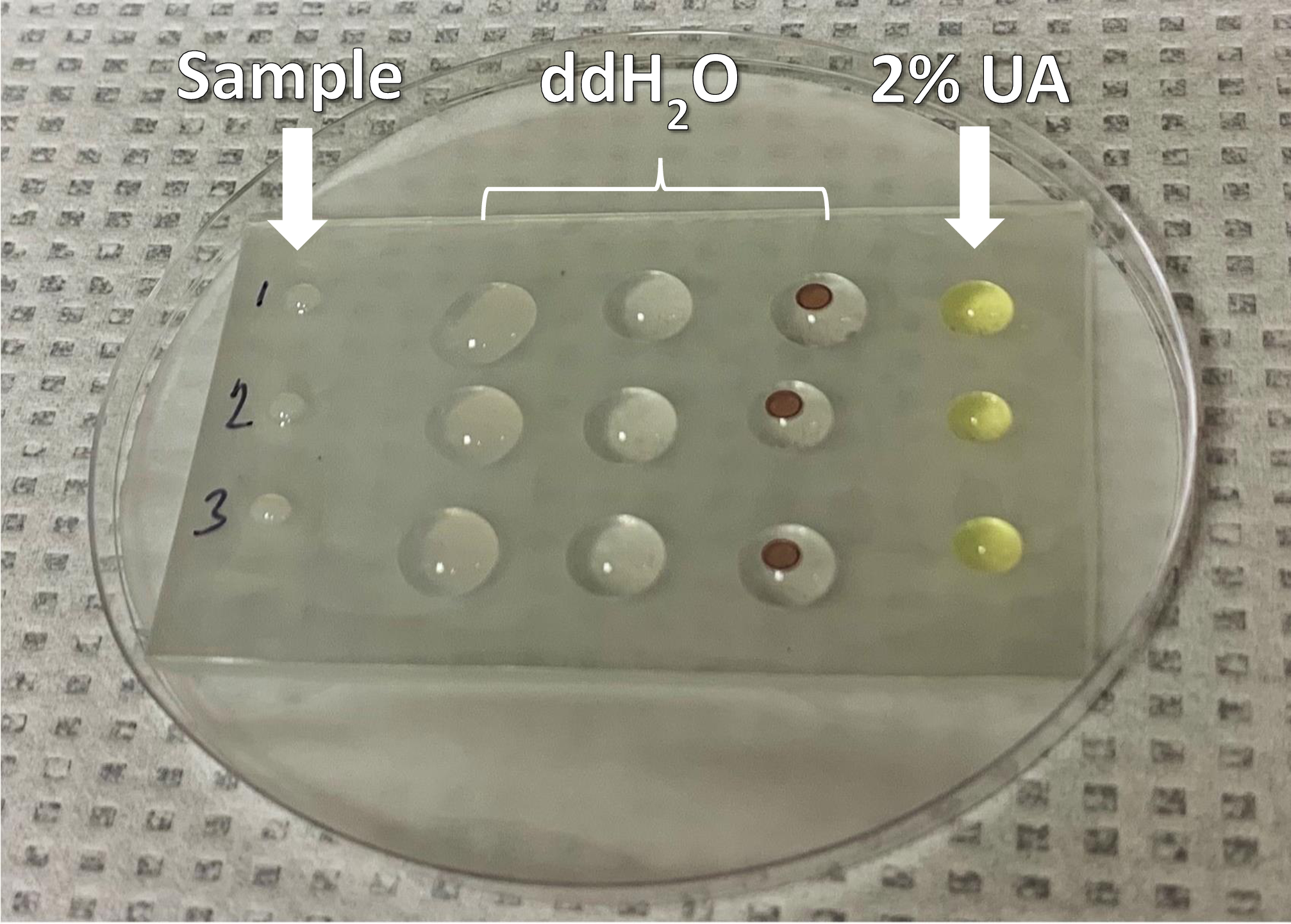

Supplement: S1 Fig — Copper grids were negatively stained with 2% uranyl acetate (UA). (TIF) [file pone.0257633.s001.tif]

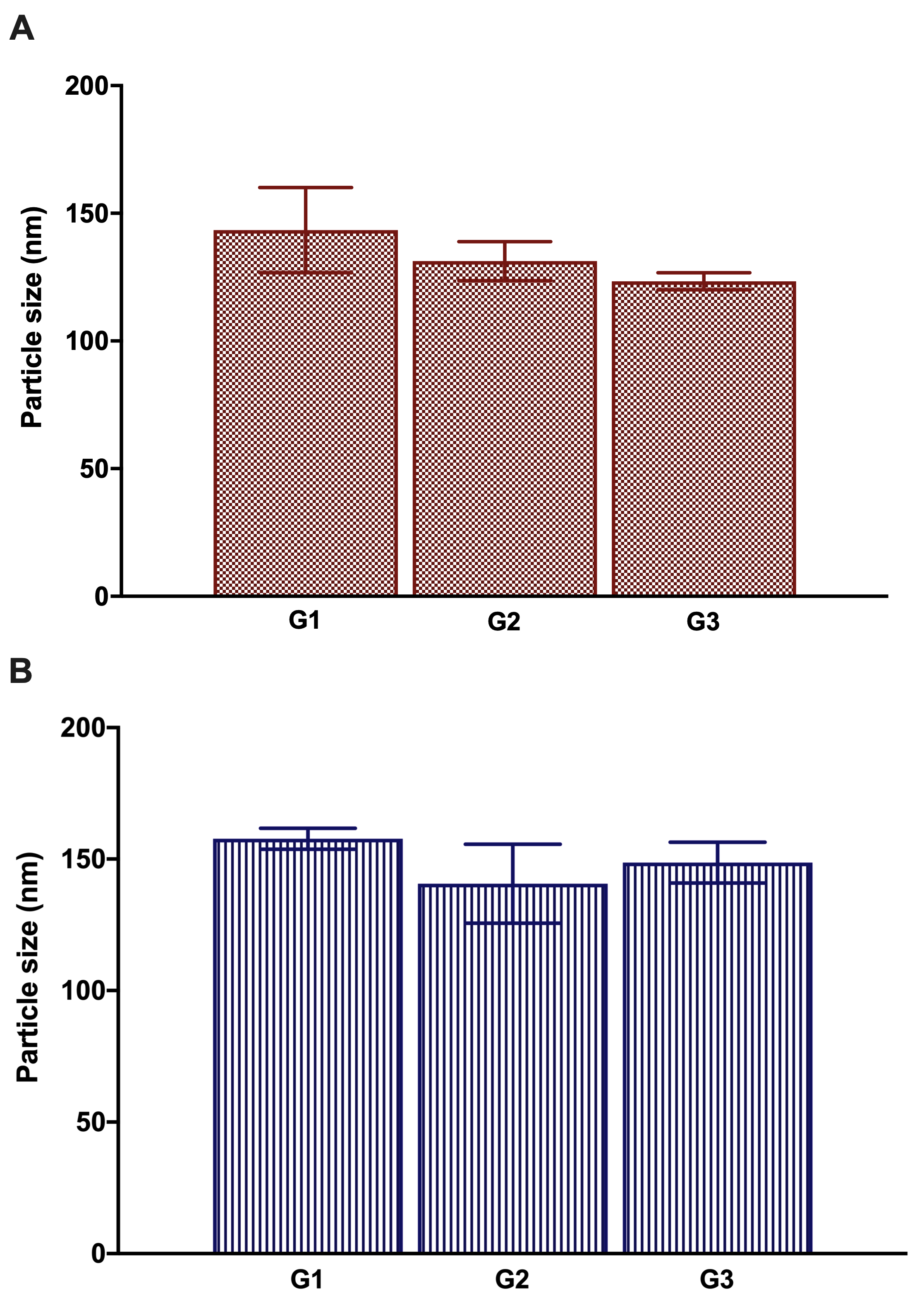

Supplement: S2 Fig — ExoQuick (A) and Differential Ultracentrifugation (B) as determined by Nanoparticle Tracking Analysis (NTA). Group (G)1: whole milk frozen immediately upon collection and processed post-thaw. G2: Whole milk processed to remove fat globules and cream prior to ultracold storage. G3: Whole milk processed to remove fat globules, cream, milk cells, and casein proteins prior to ultracold storage. Mean particle size is based on Stokes-Einstein equation with a 1:700 dilution in 1X-filtered PBS, 2 independent runs with 3 technical replicates of 30 s capture/run. (TIF) [file pone.0257633.s002.tif]

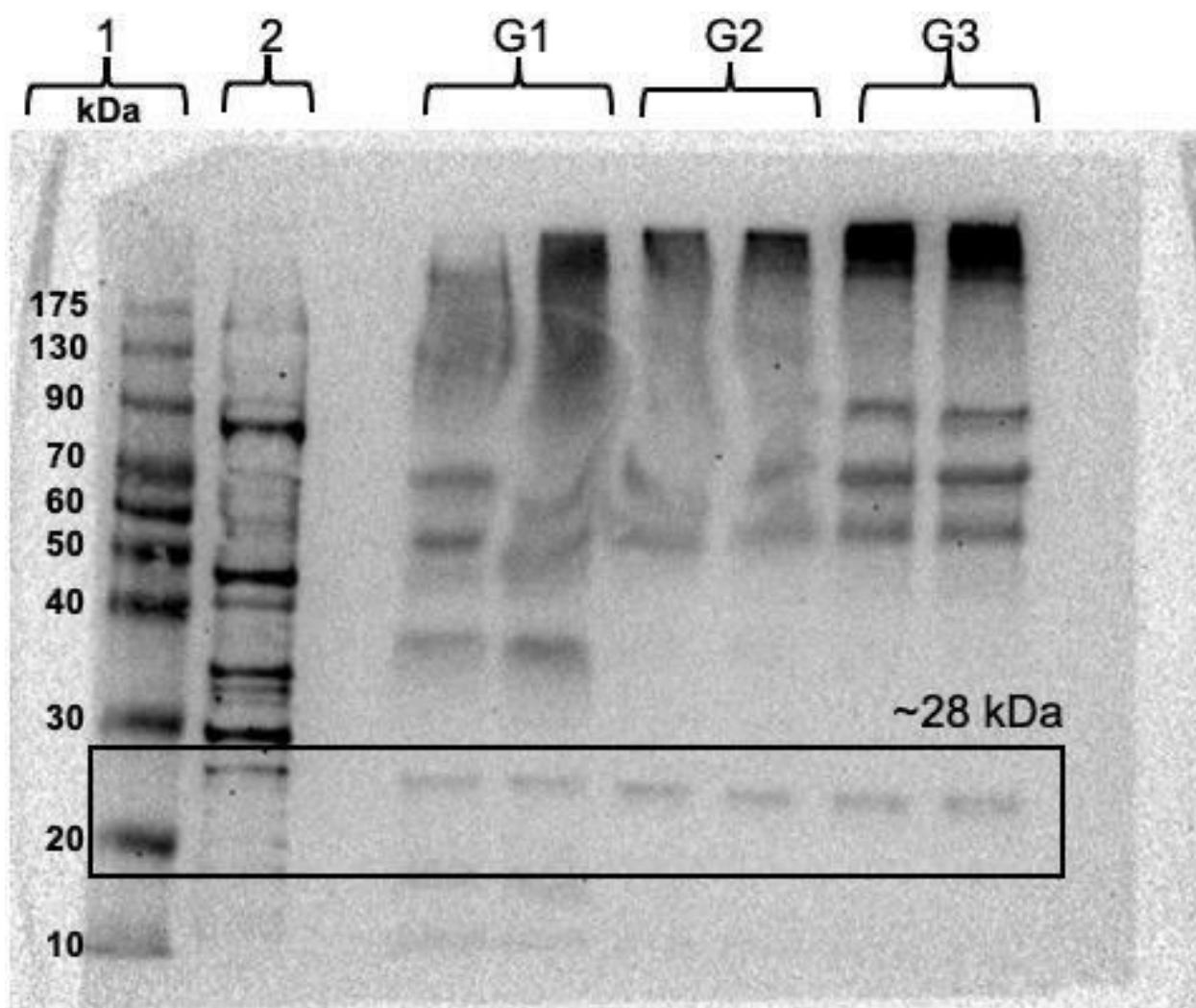

Supplementary Figure 3.

Supplement: S3 Fig — CD9 is presented as a positive, exosome marker and approximately 28 kDa in size. Lane 1: 1kB Pink Plus prestained protein ladder (range 10.5–175 kDa). Lane 2: Total soluble protein from human microglia (ATCC: HMC3 Cell line), used as the cellular control. Group 1: Frozen whole milk prior to processing. Group 2: Frozen milk without fat globules and cream. Group 3: Frozen whey fraction without fat globules, cream and casein proteins. (PDF) [file pone.0257633.s003.pdf]

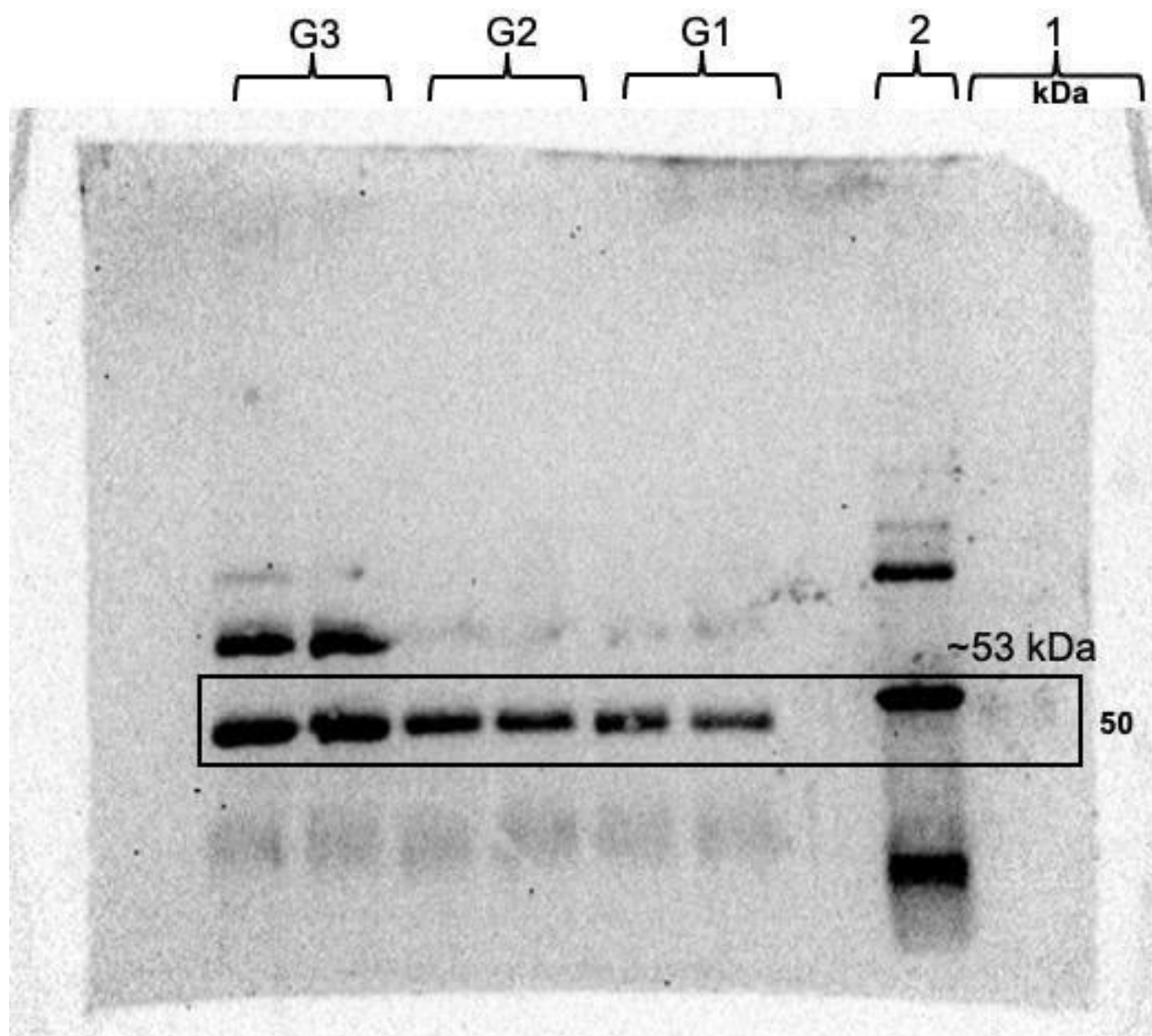

**Supplementary Figure 4.**

Supplement: S4 Fig — CD63 is presented as a positive, exosome marker and approximately 53 kDa in size. Lane 1: 1kB Pink Plus pre-stained protein ladder (range 10.5–175 kDa). Lane 2: Total soluble protein from human microglia (ATCC: HMC3 Cell line), used as the cellular control. Group 1: Frozen whole milk prior to processing. Group 2: Frozen milk without fat globules and cream. Group 3: Frozen whey fraction without fat globules, cream and casein proteins. (PDF) [file pone.0257633.s004.pdf]

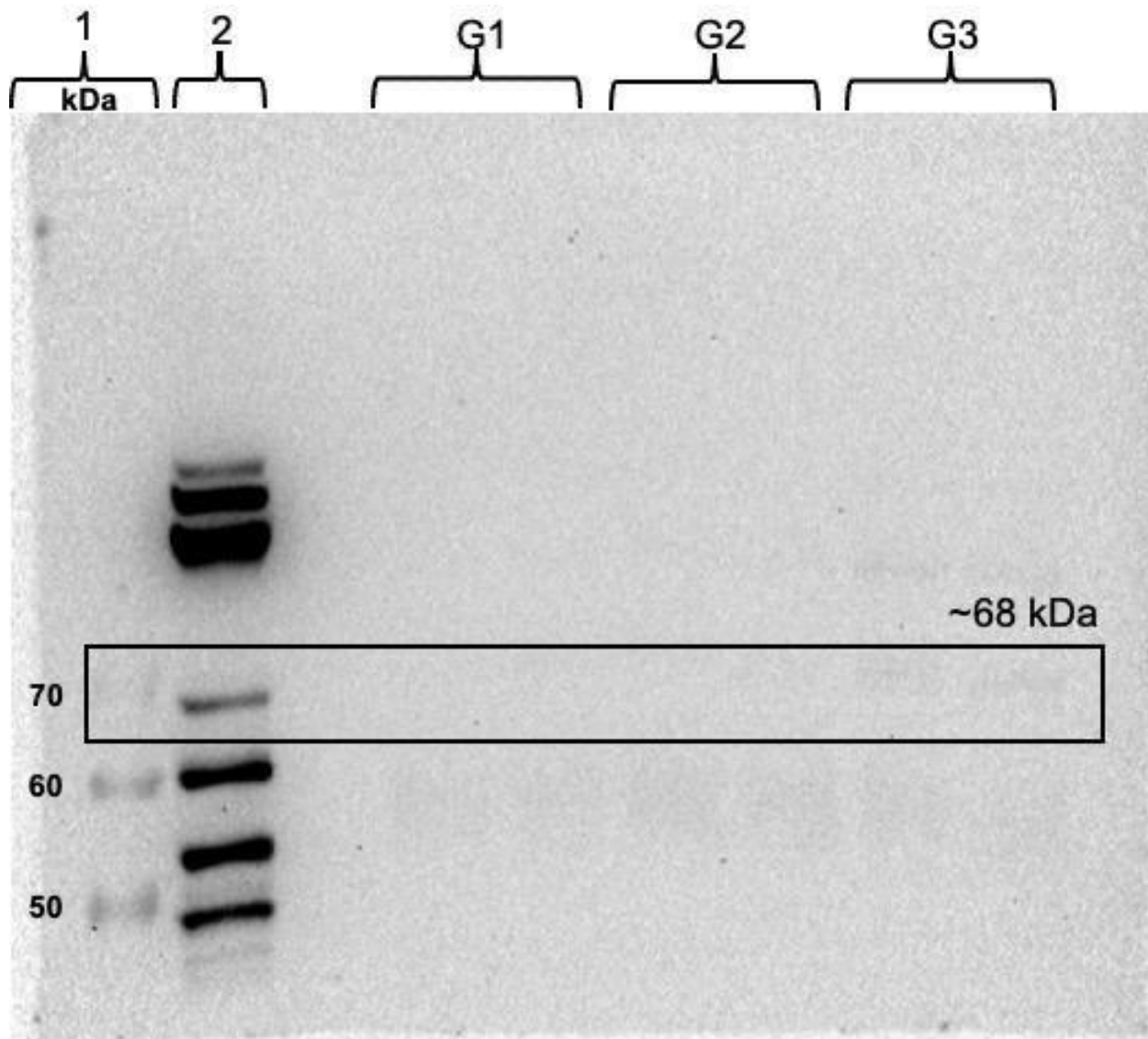

Supplementary Figure 5.

Supplement: S5 Fig — Calnexin is presented as a negative, cellular marker. Lane 1: 1kB Pink Plus prestained protein ladder (range 10.5–175 kDa). Lane 2: Total soluble protein from human microglia (ATCC: HMC3 Cell line), used as the cellular control. Group 1: Frozen whole milk prior to processing. Group 2: Frozen milk without fat globules and cream. Group 3: Frozen whey fraction without fat globules, cream, and casein proteins. (PDF) [file pone.0257633.s005.pdf]

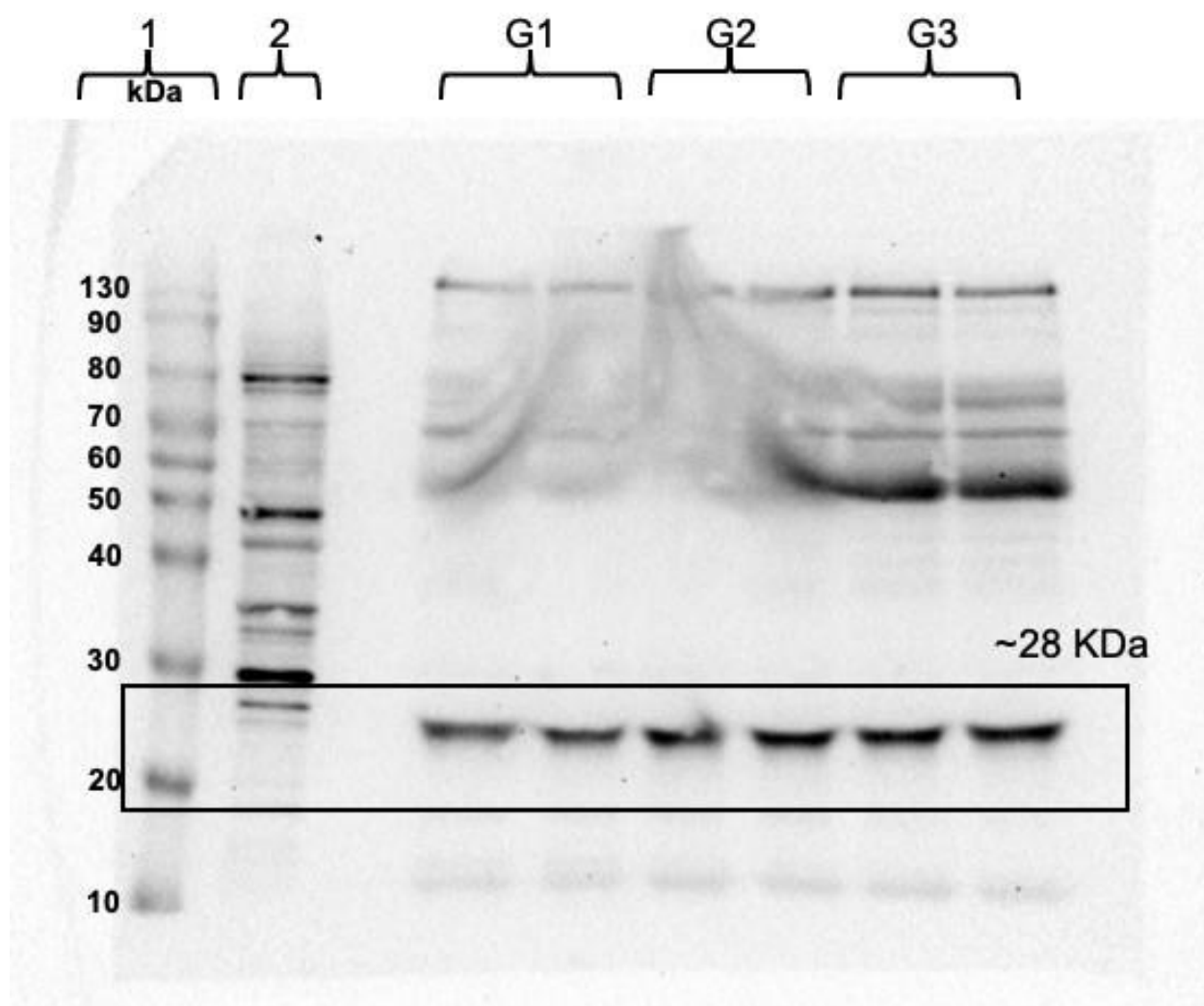

Supplementary Figure 6.

Supplement: S6 Fig — CD9 is presented as a positive, exosome marker and is approximately 28 kDa in size. Lane 1: 1kB Pink Plus pre-stained protein ladder (range 10.5–175 kDa). Lane 2: Total soluble protein from human microglia (ATCC: HMC3 Cell line), used as the cellular control. Group 1: Frozen whole milk prior to processing. Group 2: Frozen milk without fat globules and cream. Group 3: Frozen whey fraction without fat globules, cream and casein proteins. (PDF) [file pone.0257633.s006.pdf]

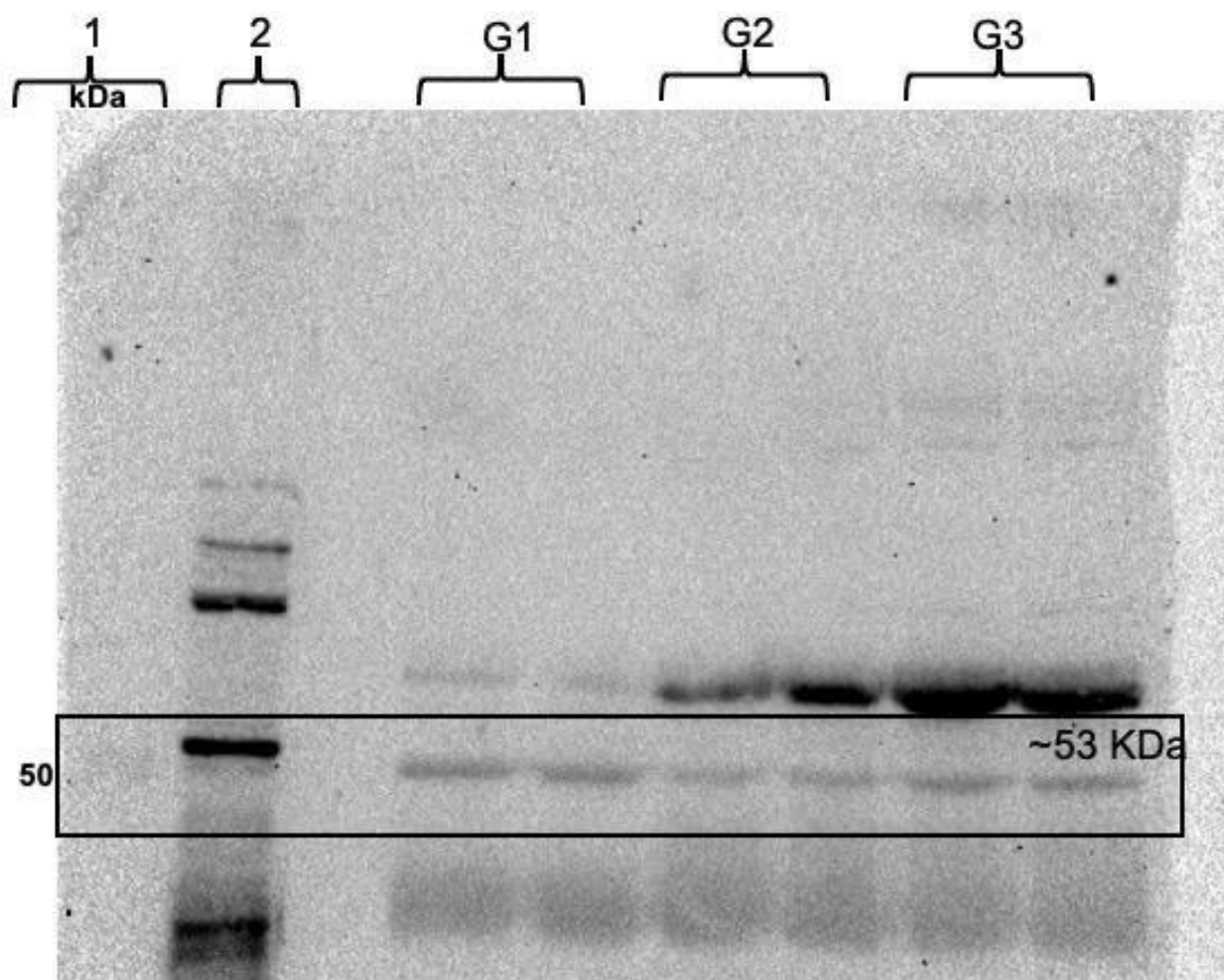

Supplementary Figure 7.

Supplement: S7 Fig — CD63 is presented as a positive, exosome marker and approximately 53 kDa in size. Lane 1: 1kB Pink Plus pre-stained protein ladder (range 10.5–175 kDa). Lane 2: Total soluble protein from human microglia (ATCC: HMC3 Cell line), used as the cellular control. Group 1: Frozen whole milk prior to processing. Group 2: Frozen milk without fat globules and cream. Group 3: Frozen whey fraction without fat globules, cream and casein proteins. (PDF) [file pone.0257633.s007.pdf]

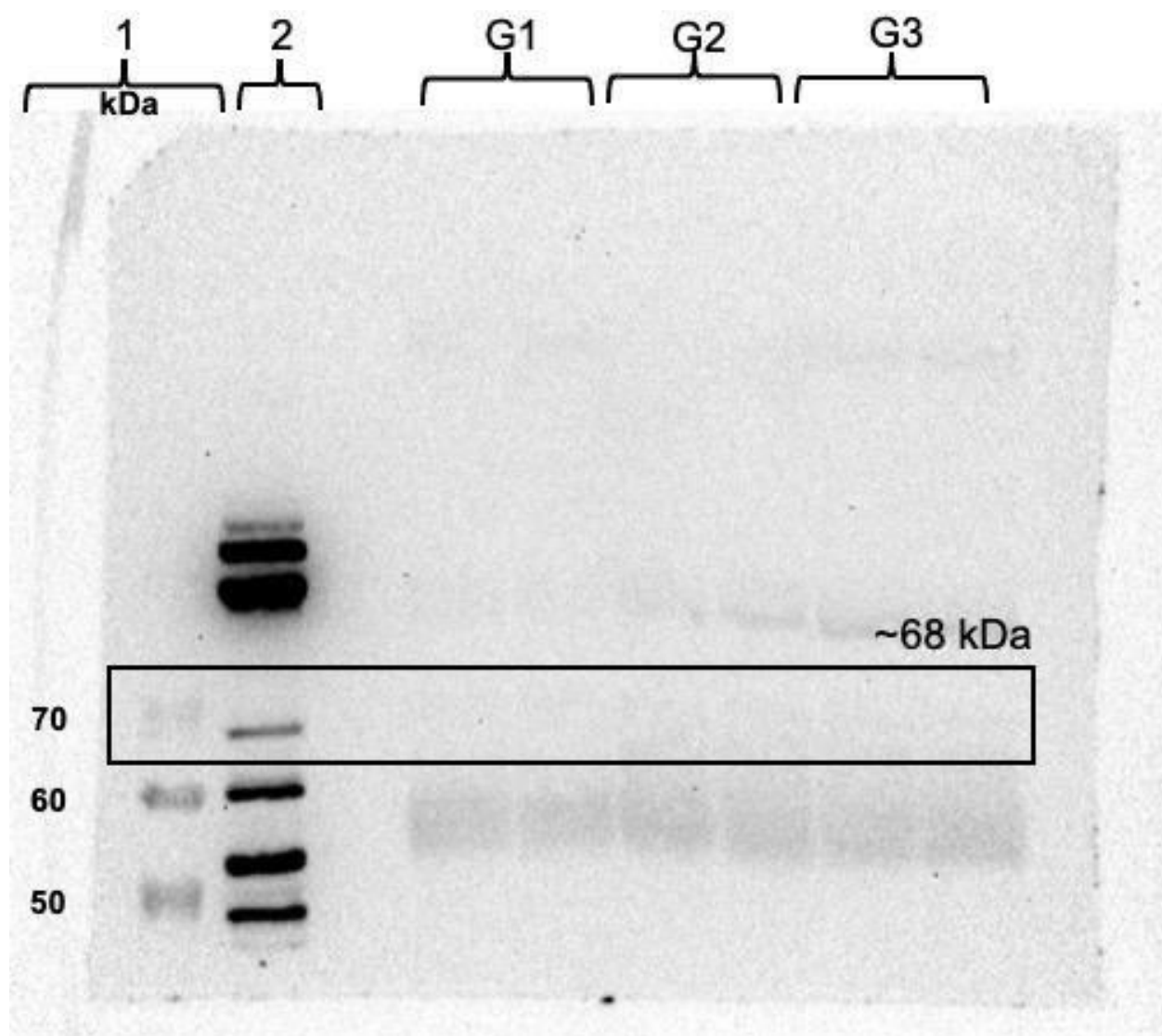

Supplementary Figure 8.

Supplement: S8 Fig — Calnexin is presented as a negative, cellular control and is approximately 68 kDa in size. Lane 1: 1kB Pink Plus pre-stained protein ladder (range 10.5–175 kDa). Lane 2: Total soluble protein from human microglia (ATCC: HMC3 Cell line), used as the cellular control. Group 1: Frozen whole milk prior to processing. Group 2: Frozen milk without fat globules and cream. Group 3: Frozen whey fraction without fat globules, cream, and casein proteins. (PDF) [file pone.0257633.s008.pdf]

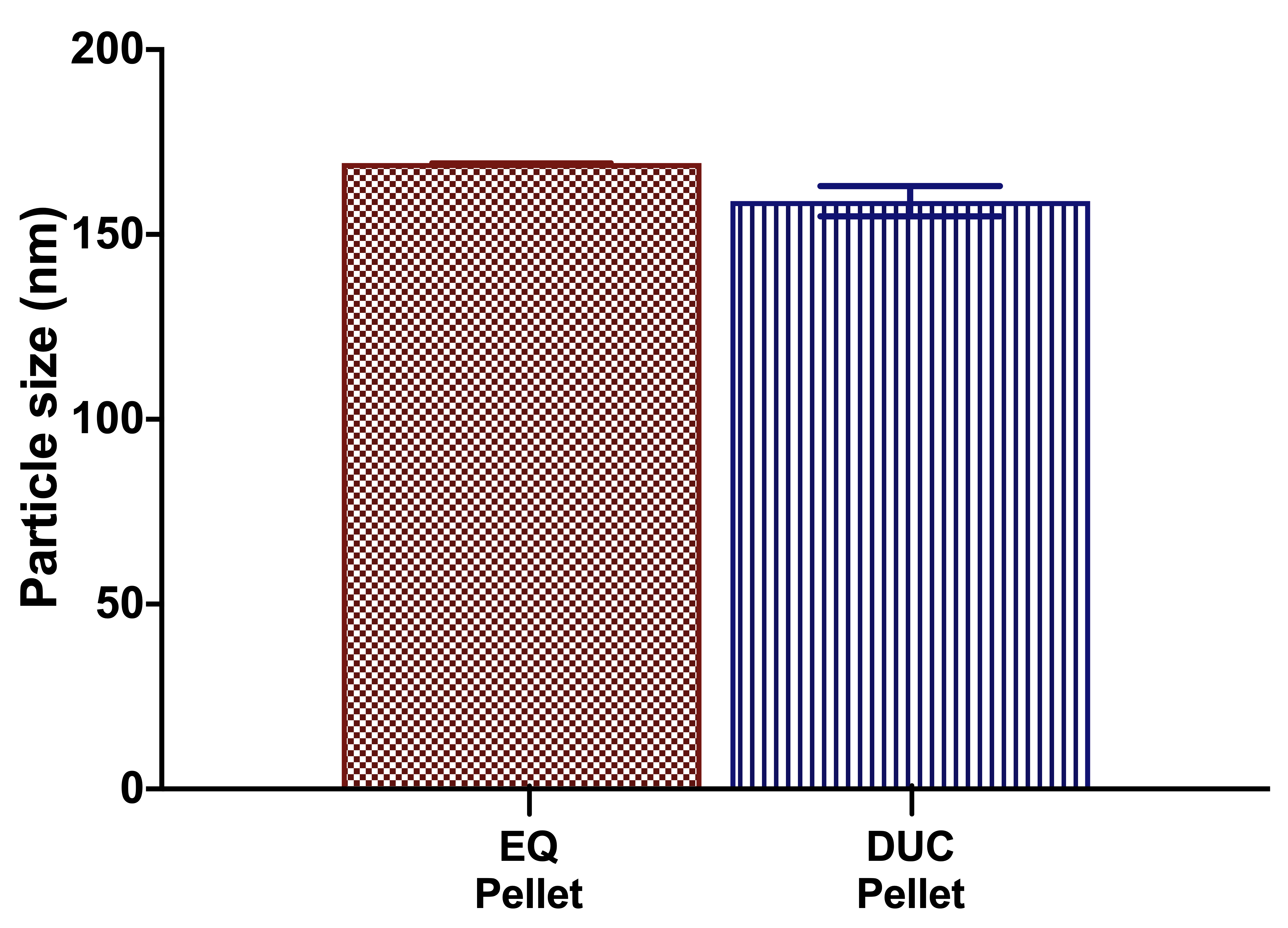

Supplement: S9 Fig — Mean particle size is based on Stokes-Einstein equation with a 1:500 dilution in 1X-filtered PBS, 2 independent runs with 3 technical replicates of 30 s capture/run. (TIF) [file pone.0257633.s009.tif]

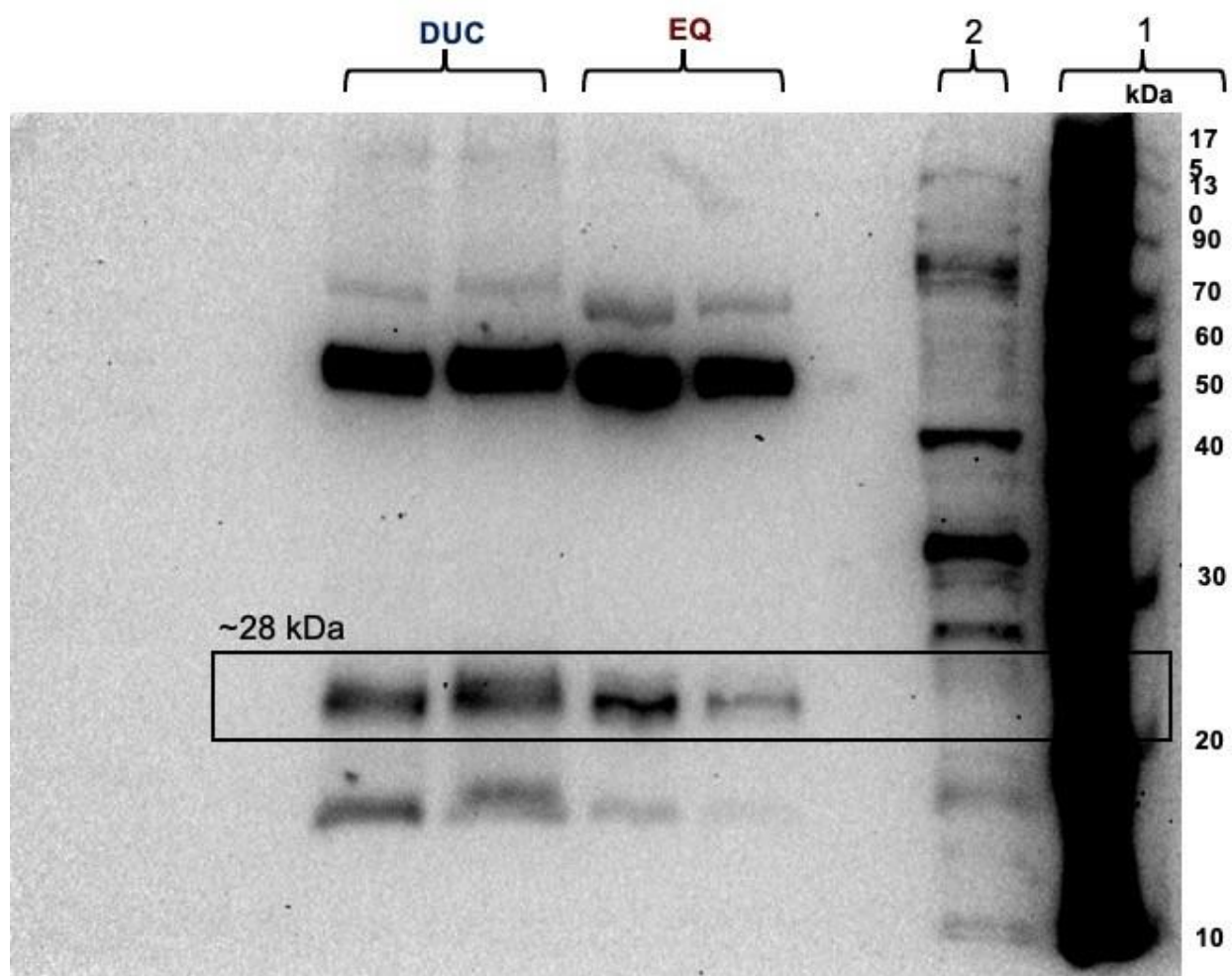

**Supplementary Figure 10.**

Supplement: S10 Fig — CD9 is presented as a positive, exosome marker and is approximately 28 kDa in size. Lane 1: 1kB Pink Plus pre-stained protein ladder (range 10.5–175 kDa). Lane 2: Total soluble protein from human microglia (ATCC: HMC3 Cell line), used as the cellular control. Lane 3–4: Milk exosomes isolated with ExoQuick protocol. Lane 5–6: milk exosomes isolated with the ultracentrifugation protocol. (PDF) [file pone.0257633.s010.pdf]

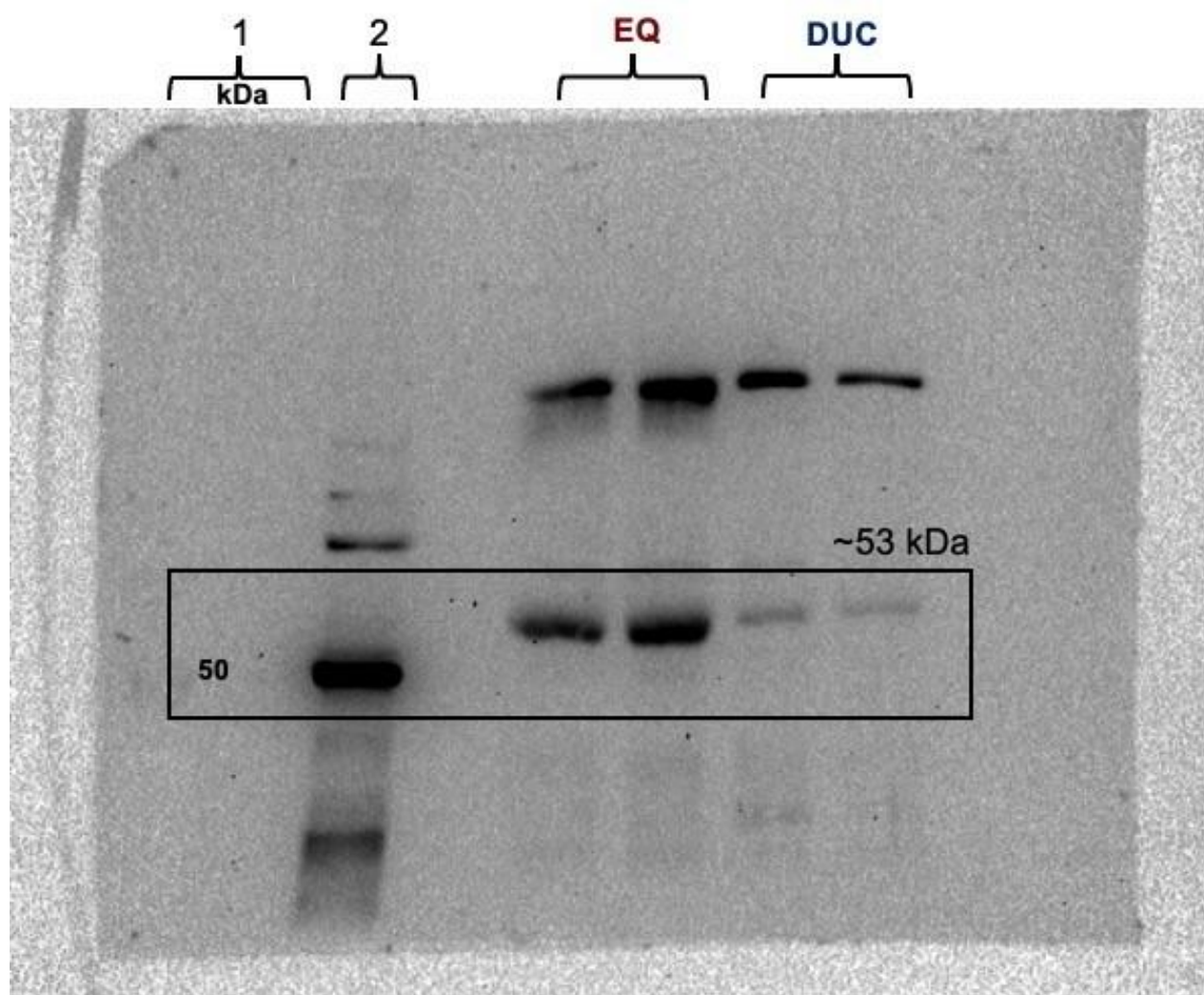

Supplementary Figure 11.

Supplement: S11 Fig — CD63 is presented as a positive, exosome marker and is approximately 53 kDa in size. Lane 1: 1kB Pink Plus pre-stained protein ladder (range 10.5–175 kDa). Lane 2: Total soluble protein from human microglia (ATCC: HMC3 Cell line), used as the cellular control. Lane 3–4: Milk exosomes isolated with ExoQuick protocol. Lane 5–6: milk exosomes isolated with the ultracentrifugation protocol. (PDF) [file pone.0257633.s011.pdf]

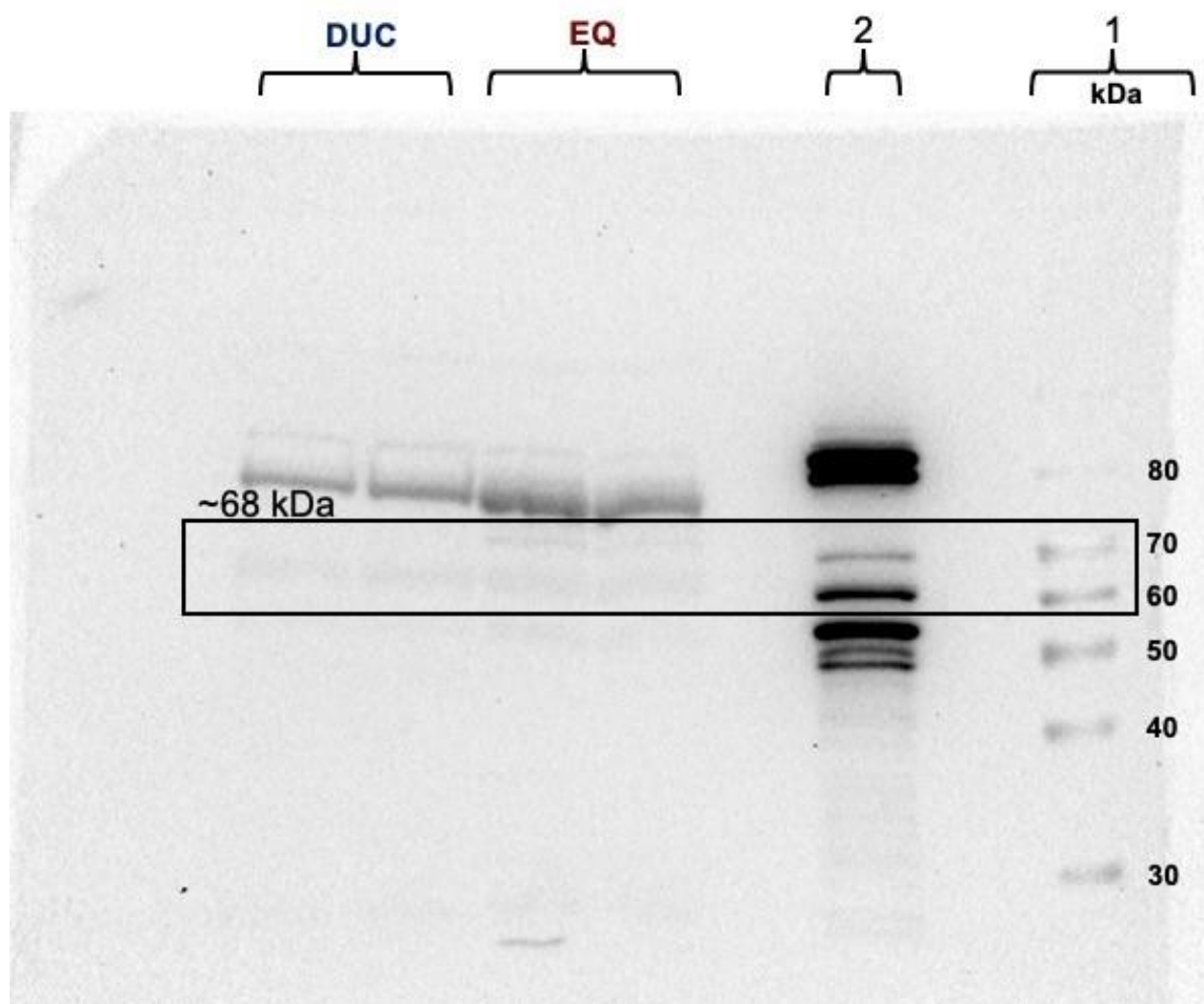

Supplementary Figure 12.

Supplement: S12 Fig — Calnexin is presented as a negative, cellular control and is approximately 68 kDa in size. Lane 1: 1kB Pink Plus pre-stained protein ladder (range 10.5–175 kDa). Lane 2: Total soluble protein from human microglia (ATCC: HMC3 Cell line), used as the cellular control. Lane 3–4: Milk exosomes isolated with ExoQuick protocol. Lane 5–6: milk exosomes isolated with the ultracentrifugation protocol. (PDF) [file pone.0257633.s012.pdf]
